# Genome-wide dissection and expression profiling of unique glyoxalase III genes in soybean reveal the differential pattern of transcriptional regulation

**DOI:** 10.1038/s41598-018-23124-9

**Published:** 2018-03-19

**Authors:** Tahmina Islam, Ajit Ghosh

**Affiliations:** 1 Plant Breeding and Biotechnology Laboratory, Department of Botany, University of Dhaka, Dhaka, 1000 Bangladesh; 2 Department of Biochemistry and Molecular Biology, Shahjalal University of Science and Technology, Sylhet, 3114 Bangladesh; 3 Department of Plant Developmental Biology, Max Planck Institute for Plant Breeding Research, Köln, 50829 Germany

## Abstract

Reactive carbonyl species, such as methylglyoxal and glyoxal are very toxic in nature and can inactivate various cellular macromolecules such as DNA, RNA, and protein by forming advanced glycation end products. Conventional glyoxalase pathway with two enzymes- glyoxalase I and glyoxalase II, detoxify MG into D-lactate with the help of reduced glutathione. However, DJ-1/PfpI domain(s) containing DJ-1/ Hsp31 proteins do the same in a single step, and thus termed as “glyoxalase III”. A comprehensive genome-wide analysis of soybean identified eleven putative glyoxalase III proteins with DJ-1/PfpI domain encoded by seven genes. Most of these proteins are predicted to be mitochondria and chloroplast localized. In spite of similar function, a differential evolution pattern was observed between Hsp31 and DJ-1 proteins. Expression of *GmDJ-1*A, *GmDJ-1*B, and *GmDJ-1*D2 transcripts was found to be constitutive in different tissues and developmental stages. Transcript profiling revealed the strong substrate-specific upregulation of *GmDJ-1* genes in response to exogenous methylglyoxal exposure. Out of seven genes, *GmDJ-1*D1 and *GmDJ-1*D2 showed maximum upregulation against salinity, dehydration, and oxidative stresses. Moreover, GmDJ-1D2 showed functional glyoxalase III enzyme activity by utilizing MG as a substrate. Overall, this study identifies some novel tissue-specific and abiotic stress-responsive *GmDJ-1* genes that could be investigated further.

## Introduction

Generation of reactive carbonyl species (RCS) is a common metabolic phenomenon of all living systems including bacteria, fungi, animals, and plants^1^. Among various RCS, α-oxoaldehyde compounds such as methylglyoxal (MG), glyoxal, phenylglyoxal and hydroxy-pyruvaldehyde are known to be highly reactive^2^. α-Oxoaldehydes are mainly produced by oxidation of glucose, lipid peroxidation, catabolism of fatty acid and amino acid, and DNA oxidation^2,3^. Due to their highly electrophilic potential^4^, α-oxoaldehydes are very reactive in nature and form adducts with the nucleophilic centers of DNA, RNA, and proteins. They can react non-enzymatically with arginine, lysine, cysteine residues of the protein^4^, and form advanced glycation end-products (AGEs). Formation of AGEs is responsible for aging and various neurodegenerative diseases of human, including diabetes, Parkinson disease, and Alzheimer disease^5^. Accumulation of excess AGEs or their precursor α-oxoaldehydes cause carbonyl stress in animals^6^ and plants^7^.

Reactive α-oxoaldehydes are detoxified by either the glutathione-dependent glyoxalase pathway^7^ or NAD[P]H-dependent aldo-keto reductase (AKR) system^8^. The major detoxification system is glyoxalase pathway that includes two enzymes- glyoxalase I (GLY I, EC 4.4.1.5) and glyoxalase II (GLY II, EC 3.1.2.6). GLY I converts hemithioacetal, a highly cytotoxic non-enzymatic adduct of MG and reduced glutathione (GSH), into S-lactoylglutathione (SLG). This SLG is further hydrolyzed into D-lactate by the action of glyoxalase II (GLY II), and one molecule of GSH is recycled back in the system (Fig. S1). The pathway was first discovered simultaneously in rabbit and dog tissues at 1913^9,10^. It has been established to be one of the most ubiquitous and evolutionary highly conserved pathways in both prokaryotic and higher eukaryotic species^2^. Expression of glyoxalase genes, as well as enzyme activity, have been reported to be altered in response to various abiotic, biotic, hormonal and chemical treatment^11^. Moreover, overexpression of MG detoxifying glyoxalase pathway provides significant abiotic stress tolerance by resisting the excess accumulation of MG in transgenic tobacco^12^ and tomato^13^ plant. Therefore, both glyoxalase enzymes and methylglyoxal level are considered as biomarkers for plant stress tolerance^11^.

Apart from this well-conserved pathway, the presence of a novel glyoxalase III (GLY III) enzyme activity has been known for a long time in *Escherichia coli* (E. coli) total cell lysates^14^. GLY III could convert MG into D-lactate in a single step without the help of any cofactor (Fig. S1). Recently, one of the *E*. *coli* gene (*hchA*) product, Hsp31was reported having *in vitro* GLY III activity^15^. Although GLY III activity of Hsp31 is very low as compared to the conventional GLY I/II^16^, it has been consistent and significant enough to study further in other species. Apart from *E*. *coli*, GSH independent GLY III activity has been reported in *Homo sapiens*, *Caenorhabditis elegans*, *Drosophila melanogaster*^5^, *Saccharomyces cerevisiae*^17^, *Schizosaccharomyces pombe*^1^, *Candida albicans*^18^, *Arabidopsis thaliana*^19^, and *Oryza sativa* L.^20^ till date. Moreover, GLY III proteins were reported to be metal independent^15^, while conventional GLY I enzyme requires either Ni^2+^ or Zn^2+^ for its optimal activity^21^.

Structural analysis of *E*. *coli* GLY III protein indicates that it is a member of DJ-1/PfpI superfamily^15^. As GLY III activity was found to reside in the DJ-1 domain containing proteins, they were named as “DJ-1” protein in various species. However, they were named as Hsp protein in *E*. *coli*, yeast, and several fungi species. In human, DJ-1 protein has been found to be associated with cancer^22^ and Parkinson’s disease^23^. Apart from dicarbonyl detoxification, DJ-1 has been reported to be involved in regulation of transcription and mitochondrial function and having a molecular chaperone and protease activity^1^. DJ-1 proteins possess a conserved catalytic triad Glu-Cys-His in the active site. Among them, the cysteine residue was found to be highly conserved and oxidation of this residue is critically required for DJ-1 catalytic activity^5^. The catalytic activity of DJ-1/Hsp31 (GLY III) protein has been reported from various organisms including *A*. *thaliana*, *O*. *sativa*, *H*. *Sapiens*, *E*, *coli*, *D*. *melanogaster*, *C*. *albicans*, *S*. *pombe*, *S*. *cerevisiae* etc^1,5,15,17–20^. Due to the lower catalytic efficiency as compared to conventional GLY enzymes^15^, concerns were raised about the GLY III activity of DJ-1^24^. Recently, the deglycation activity of DJ-1 protein has been reported as an artifact of TRIS buffer using knockdown DJ-1β *Drosophila* flies^25^. But, GLY III activity for AtDJ-1D and OsDJ-1C has been reported using sodium phosphate buffer without such influence^19,20^. Moreover, *E*. *coli* Hsp31 (EcHsp31) serves as a heat-inducible molecular chaperone and provides protection against heat starvation and oxidative stresses, too^26^. Thus, the exact mechanism of how DJ-1 executes multiple cellular functions is somewhat unclear.

Previously, genome-wide analysis of DJ-1 gene has been carried out in *Arabidopsis thaliana* (L.) Heynh., *Oryza sativa* L. and *Medicago truncatula* L.; and identified six, six and five *DJ-1* genes in respective genomes that code for eleven, twelve and six proteins, respectively^7,20,27^. Among *Arabidopsis* members, AtDJ-1d showed highest GLY III activity^19^, while the GLY III activity of OsDJ-1C has been shown experimentally^20^. Different AtDJ-1, OsDJ-1, MtDJ-1 members showed a different pattern of sub-cellular localization indicating their important role in various organelles. All *OsDJ-1* genes showed substrate (MG) and oxidative stress induced transcript up-regulation^20^. It has been reported that loss-of-function of AtDJ-1a induces cell death^28^ and knockout of AtDJ-1c lead to non-viable albino seedling generation in *Arabidopsis*^29^. Expression of *MtDJ-1*A and *MtDJ-1*D found to be highly up-regulated in response to drought stress^27^. All these studies prompted us to do further research on this novel pathway to unravel their role in plants. In the present study, a database based search was performed to identify DJ-1 proteins in soybean (*Glycine max*). A sequence homology-based phylogenetic analysis was performed among different GLY III proteins of both prokaryotic and eukaryotic lineages. Further, expression of newly identified members was analyzed in various tissues, and developmental stages as well as in response to adverse environmental conditions using publicly available microarray data and RT-PCR study. These analyses will provide the initial clues to understand the role of DJ-1 protein family in the field of stress physiology and cell biology.

## Results

### *In silico* analysis identified eleven putative DJ-1 proteins in soybean

**Figure 1:**
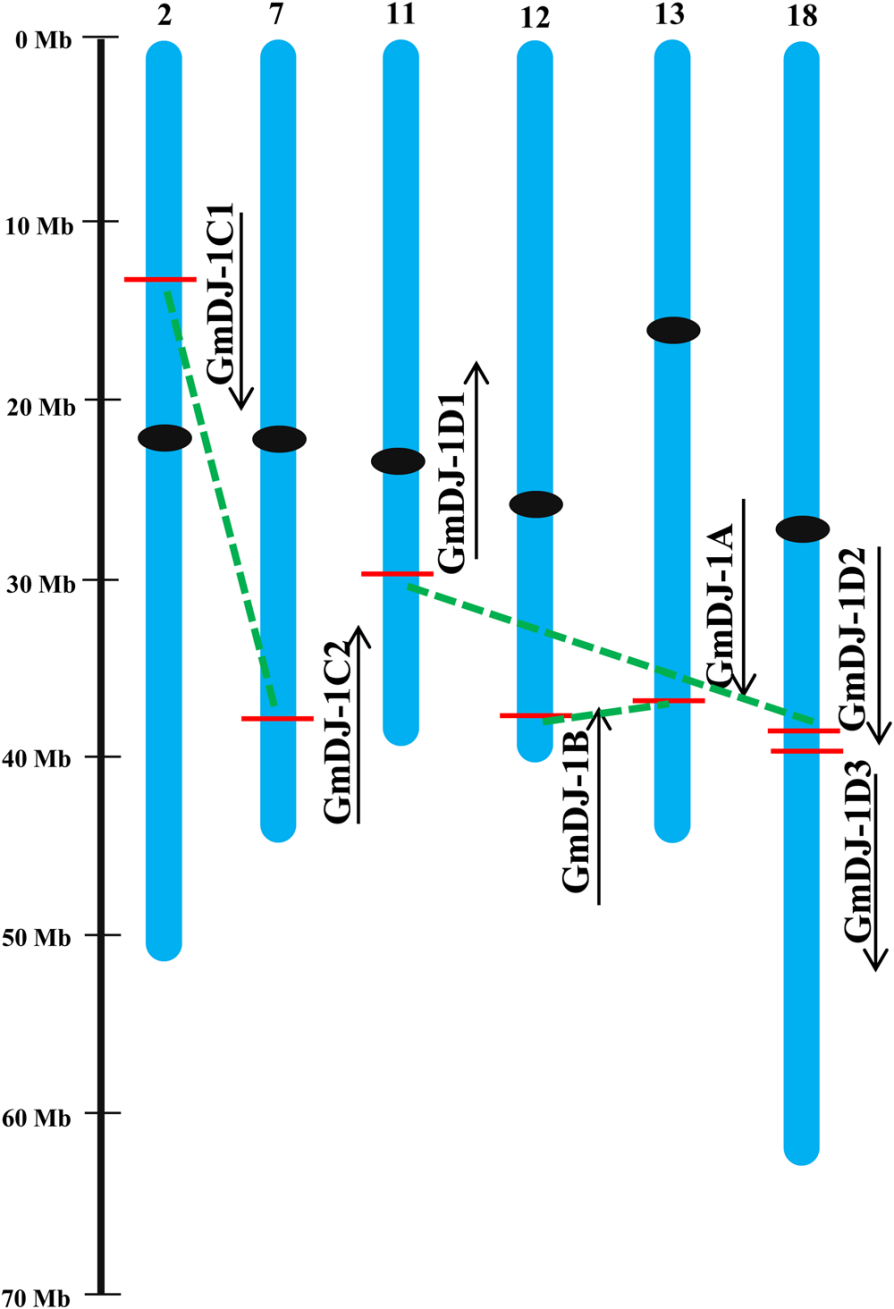
Chromosomal distribution and orientation of Soybean *Glyoxalase III* (*GmDJ-1*) genes. The position of the newly identified Glyoxalase III genes (*DJ-1*, A-D) has been identified and marked in different chromosomes of soybean. Only six soybean chromosomes (2, 7, 11, 12, 13, and 18) shown in the Fig. that has *DJ-1* genes out of twenty in total. Chromosome numbers are indicated at the top of each bar, centromeres are pointed by a black circle and relative size of the chromosomes are indicated by the scale at the left. Duplicated genes are joined by a green dashed line.

A total of eleven DJ-1 proteins were identified in soybean genome (Table 1) which is almost similar to the previously reported *Arabidopsis* (11) and rice (12) DJ-1 protein number^20^. The newly identified members were nomenclature based on their *Arabidopsis* orthologs as proposed previously^30^ (Table 1). These eleven proteins were found to be coded by seven unique *DJ-1* genes and located on six different chromosomes of soybean namely 2, 7, 11, 12, 13, and 18 (Fig. 1). Two genes resided on chromosome no 18, and rest of the chromosome has only one gene each (Fig. 1). Chromosomes without any *DJ-1* genes were not present in Fig. 1. Gene duplication analysis demonstrated three duplication events- *GmDJ-1*C1/C2, *GmDJ-1*D1/D2, and *GmDJ-1*B/A (Fig. 1, Table S1). Based on nonsynonymous substitutions (Ka) and synonymous substitutions (Ks) of each gene pair, the evolutionary history of selection acting on different genes could be measured^31,32^. The Ka/Ks of three *GmDJ-1* duplicated gene pairs (Table S1) was found to be less than 0.55; indicates the influence of purifying selection in the evolution of the gene pairs. Considering the divergence rate of 6.161029 synonymous mutations per synonymous site per year for soybean^33^, the duplicated pairs showed a divergence time frame between 9.03 to 11.17 Mya (Table S1). Apart from paralogous gene duplications, GmDJ−1 genes were analyzed further to identify the orthologous gene duplication events in three plants (*Arabidopsis*, Rice, and *Medicago*) using plant genome duplication database (http://chibba.agtec.uga.edu/duplication/index/downloads)^34^. This analysis revealed the presence of three *AtDJ*−1, one *OsDJ*−1 and three *MtDJ*-1 duplicated genes with GmDJ-1 family (Table 2). All these orthologous gene pairs showed the Ka/Ks ratio of less than 1; indicating the effect of purifying selection in the evolution of DJ-1 genes among *Arabidopsis*, rice, *Medicago*, and soybean.

**Table 1:** List of putative *DJ-1* genes in Soybean (*Glycine max*) along with their chromosomal locations, alternatively spliced forms, coding DNA sequence (CDS), polypeptide (PP) length, molecular weight (MW), isoelectric point (pI) and localization (bp base pair, aa amino acid, kDa kilodalton).

Name	Gene	Protein	CDS coordinate (5′ to 3′)	Length		MW (kDa)	pI	Localization
				CDS (bp)	PP (aa)			
GmDJ-1C1	Glyma.02G131600	Glyma.02g131600.1	13577916–13581914	930	309	33.5	9.27	Mt^a,b^; Ch^b^
		Glyma.02g131600.2		819	272	29.6	7.77	Mt^a,b^; Cy^a,b^
		Glyma.02g131600.3		801	266	29.0	8.79	Mt^a,b^
GmDJ-1C2	Glyma.07G213200	Glyma.07g213200.1	38539847–38544732	1353	450	48.1	9.15	Mt^a^; Ch^b,c^
		Glyma.07g213200.2		1347	448	47.8	9.15	Mt^a^; Ch^b,c^
GmDJ-1D1	Glyma.11G207900	Glyma.11G207900.1	29722467–29727693	912	303	32.4	5.01	Pm^a,b^
GmDJ-1B	Glyma.12G228600	Glyma.12G228600.1	38853910–38857557	1308	435	46.6	6.34	Ch^a,b,c^
GmDJ-1A	Glyma.13G271200	Glyma.13G271200.1	37329145–37332717	1314	437	46.8	6.48	Ch^a,b,c^
GmDJ-1D2	Glyma.18G045900	Glyma.18G045900.1	3971126–3974924	1164	387	41.1	5.34	Cy^a,b^; Ch^b^
		Glyma.18G045900.2		990	329	35.0	5.24	Cy^a,b^; Ch^b^
GmDJ-1D3	Glyma.18G046000	Glyma.18G046000.1	3976764–3980543	1164	387	41.7	5.25	Cy^a,b^

Abbreviations: Ch chloroplast, Cy cytosol, Mt mitochondria, Pm plasma membrane.

^a^Localization prediction by CELLO v.2.5 (http://cello.life.nctu.edu.tw/).

^b^Localization prediction by pSORT (http://wolfpsort.org/).

^c^Chloroplast localization signal confirmed by ChloroP (http://www.cbs.dtu.dk/services/ChloroP/).

**Table 2:** List of orthologous GmDJ-1 genes present in *Arabidopsis thaliana*, *Oryza sativa*, and *Medicago truncatula*.

	*Arabidopsis thaliana*				*Oryza sativa*				*Medicago truncatula*			
Locus 1	Locus 2	Ka	Ks	Ka/Ks	Locus 2	Ka	Ks	Ka/Ks	Locus 2	Ka	Ks	Ka/Ks
GmDJ-A	AtDJ-1A	0.2866	23.399	0.01	—	—	—	—	MtDJ-1A	0.1087	0.534	0.20
GmDJ-B	AtDJ-1B	0.266	22.824	0.01	OsDJ-1A	0.3614	0.0	∞	MtDJ-1A	0.1057	0.5288	0.20
GmDJ-1C1	—	—	—	—	—	—	—	—	MtDJ-1E	0.1213	0.475	0.25
GmDJ-1C2	AtDJ-1C	0.3224	16.139	0.02	—	—	—	—	MtDJ-1E	0.1265	0.6459	0.19
GmDJ-1D1	—	—	—	—	—	—	—	—	MtDJ-1B	0.4826	0.8013	0.60
GmDJ-1D2	—	—	—	—	—	—	—	—	MtDJ-1B	0.3963	0.6846	0.58
GmDJ-1D3	—	—	—	—	—	—	—	—	—	—	—	—

### Identified GmDJ-1 members showed great variation in their structure

As eleven GmDJ-1 proteins are coded by seven genes, indicate the presence of alternative splicing event. Only three out of seven *GmDJ-1* genes (*GmDJ-1*C1, *GmDJ-1*C2, and *GmDJ-1*D2) showed alternative splicing (Fig. S2). *GmDJ-1*C1 generates three transcripts, while *GmDJ-1*C2 *and GmDJ-1*D2 generate two each (Fig. S2, Table 1). All these transcripts have minimum 4 (*GmDJ-1*D2.2) to maximum 8 (*GmDJ-1*D1) exons, with 7 exons in maximum four transcripts (*GmDJ-1*C2.1, *GmDJ-1*C2.2, *GmDJ-1*B, *GmDJ-1*A) (Table S2). All these transcripts vary in their size from lowest 801 bp (*GmDJ-1*C1.3) to highest 1353 bp (*GmDJ-1*C2.1) with an average of 1100 bp. Similarly, polypeptide length of all these eleven proteins varies from 266 amino acids (GmDJ-1C1.3) to 450 amino acids (GmDJ-1C2.1) with an average of 365 amino acids. Consequently, eleven GmDJ-1 proteins have an average molecular weight of ~40 kDa (39.3 kDa precisely) (Table 1). In terms of isoelectric point (pI) value, proteins showed equal distribution between acidic and basic nature. Five out of eleven (GmDJ-1C1.1, GmDJ-1C1.2, GmDJ-1C1.3, GmDJ-1C2.1 and GmDJ-1C2.2) showed basic isoelectric point (pI) value (more than 7), whereas rest six (GmDJ-1D1, GmDJ-1B, GmDJ-1A, GmDJ-1D2.1, GmDJ-1D2.2 and GmDJ-1D3) showed acidic pI value (Table 1). This ensures the coexistence of both positively and negatively charged GmDJ-1 proteins at neutral physiological pH (~7.2). In terms of protein architecture, all GmDJ-1 proteins were found to have two DJ-1/PfpI domains except for GmDJ-1C1.2 and GmDJ-1C1.3 with a single domain (Fig. S2). Chloroplast localization of GmDJ-1B and GmDJ-1A proteins were predicted by three independent analysis tools (Table 1), followed by GmDJ-1C2.1 and GmDJ-1C2.2 were confirmed by pSORT and ChloroP, while GmDJ-1C1.1, GmDJ-1D2.1, and GmDJ-1D2.2 were predicted by only pSORT (Table 1). Similarly, cytosolic localization of GmDJ-1C1.2, GmDJ-1D2.1, GmDJ-1D2.2, and GmDJ-1D3; and mitochondrial localization of GmDJ-1C1.1, GmDJ-1C1.2, and GmDJ-1C1.3 were confirmed by both CELLO and pSORT (Table 1). Only one protein, GmDJ-1D1 was predicted to be localized in the plasma membrane according to both CELLO and pSORT (Table 1).

### Evolutionary GLY III proteins are found to be highly diverged

**Figure 2:**
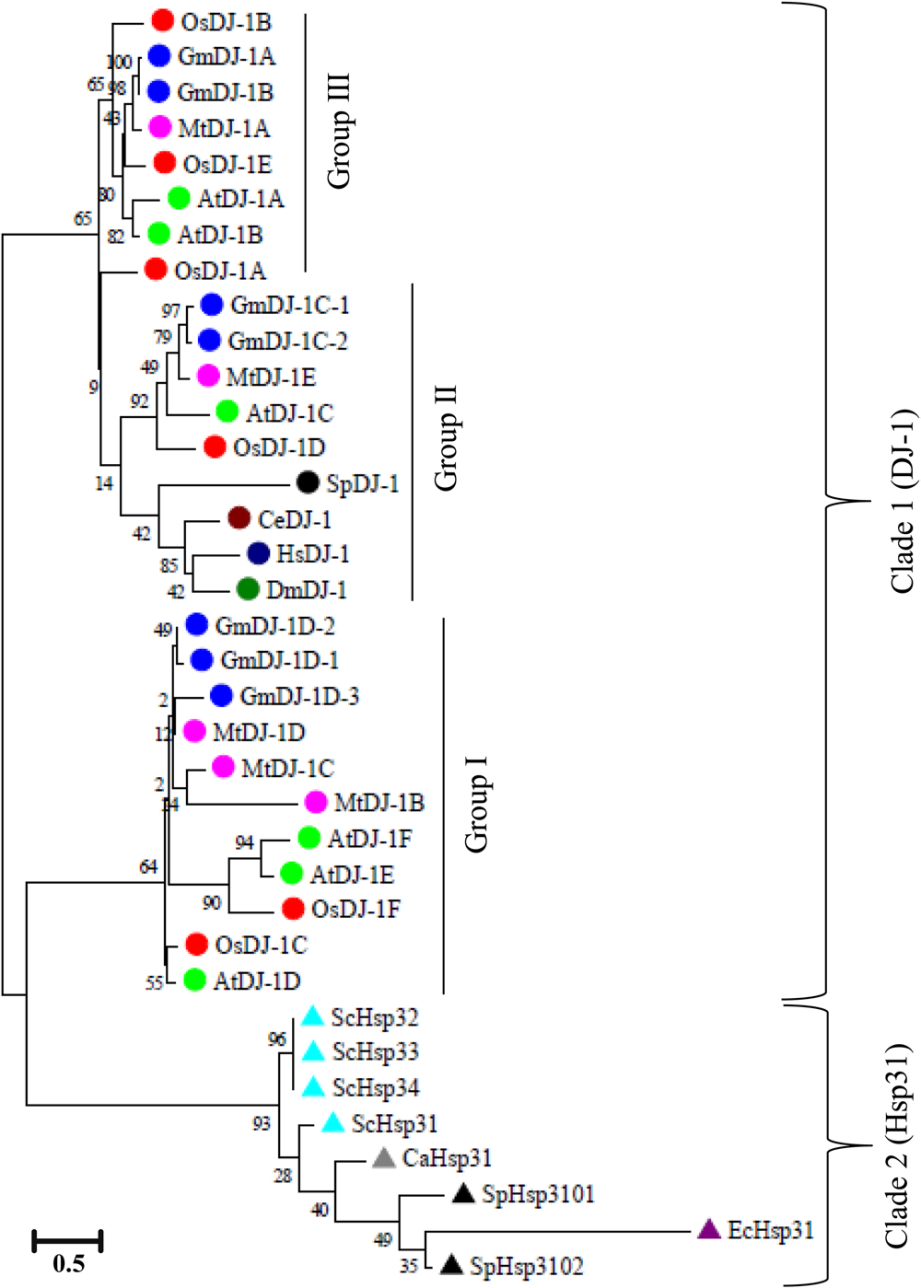
Phylogenetic analysis of GmDJ-1 with other characterized GLY III proteins. The phylogenetic tree was constructed based on multiple sequence alignments of GmDJ-1 family, AtDJ-1 family, MtDJ-1 family, OsDJ-1 family, *H*. *sapiens* DJ-1 (HsDJ-1), *D*. *melanogaster* DJ-1 (DmDJ-1), *C*. *elegans* DJ-1 (CeDJ-1.1 and CeDJ-1.2), *S*. *pombe* (SpDJ-1), *E*. *coli* Hsp31 (EcHsp31), *S*. *cerevisiae* Hsp (ScHsp31, ScHsp32, ScHsp33, ScHsp34), *C*. *albicans* Hsp31 (CaHsp31), *S*. *pombe* Hsp (SpHsp3101 and SpHsp3102) proteins. The sequences were aligned using clustalW and best tree model was predicted using Prottest 2.4 server analysis (http://darwin.uvigo.es/software/prottest2_server.html). The tree was constructed using Mega 7.0 based on Whelan and Goldman ( + freq.) model with five distinct Gamma distributed invariant sites (G + I) with 90% partial deletion and 500 bootstraps. Two major clades were denoted as Clade-I (DJ-1 proteins) and Clade-II (Hsp31 proteins). The bootstrap values are indicated by the exact number in each branch point.

To evaluate the evolutionary relationship of GLY III proteins among various species, two different types of GLY III activity providing protein such as DJ-1 and Hsp31 sequences were considered. In addition to the newly identified GmDJ-1 proteins; DJ-1 proteins from *Arabidopsis*, rice, *Medicago*, *Homo sapiens*, *Drosophila melanogaster* and *Schizosaccharomyces pombe*; and Hsp31 proteins from *E*. *coli*, *S*. *cerevisiae*, *Candida albicans* and *S*. *pombe* were used for the analysis. The analysis considered a total 38 protein sequences from different species. These protein sequences were initially analyzed using Prottest 2.4 server to identify the best fitted phylogenetic tree model (Text S1). The analysis indicates that Whelan and Goldman (+freq.) model with invariant sites (G + I) rates is the best model for these sequences. An unrooted phylogenetic tree was built based on this model with partial deletion of 90% site coverage and 500 bootstraps (Fig. 2). Two distinct clades were identified; all Hsp proteins of bacteria, yeast, and fungi form a single cluster; while DJ-1 proteins were found to be clustered in a separate clade (Fig. 2). The presence of close relationship among all individual protein class indicates a diverse evolutionary pattern of Hsp31 and DJ-1 proteins. Clade 1 (DJ-1) could be further subdivided into three groups, namely Group I, Group II and Group III. Among them, the group I and III contain only four plant DJ-1 proteins (rice, *Arabidopsis*, *Medicago*, and soybean), while group II contains proteins from both plant and non-plant sources (Fig. 2). Plant DJ-1 proteins might have some distinct characteristics that make them separated from non-plant counterparts. Experimentally characterized active GLY III member of rice, OsDJ-1C, and enzymatically most active *Arabidopsis* GLY III, AtDJ-1D are found to be present in the same group I along with another one rice, two *Arabidopsis*, three *Medicago* members, and three soybean members (GmDJ-1D1, GmDJ-1D2, and GmDJ-1D3). However, comparatively less active GLY III members of *Arabidopsis* such as AtDJ-1A and AtDJ-1B were found to form a separate group (III) with three rice, one *Medicago*, and two soybean DJ-1 members (GmDJ-1A and GmDJ-1B). However, one member of each plant species formed another group (II) with enzymatically highly active non-plant DJ-1 members. Thus, there is a possibility of fluctuation in GLY III enzyme activity for all plant DJ-1 proteins.

### GmDJ-1 proteins possess all the conserved residues of active GLY III enzyme

**Figure 3:**
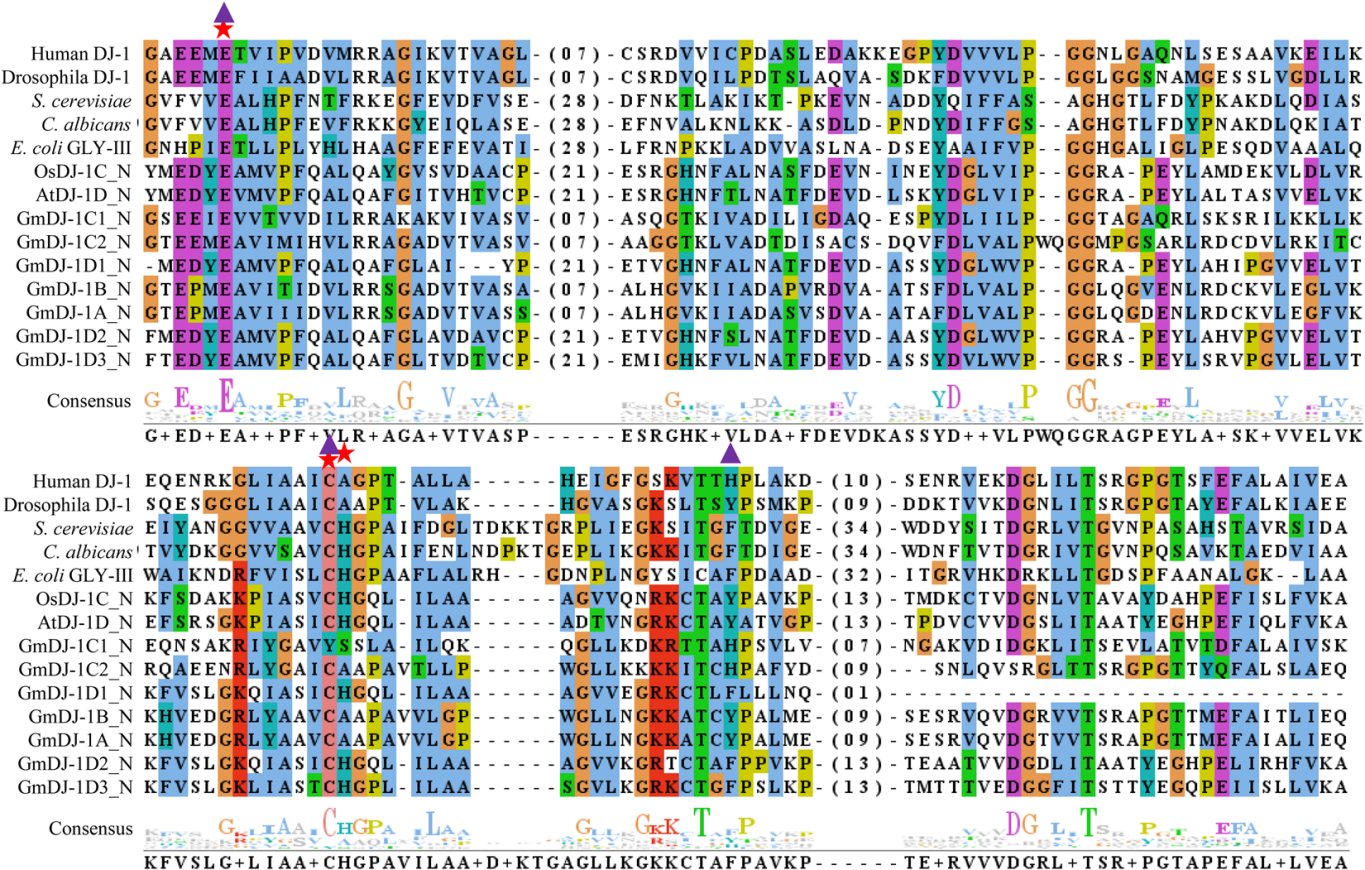
Multiple sequence alignment of GmDJ-1 proteins with other characterized GLY III proteins from various species. Putative N-terminal DJ-1/PfpI domain of all seven GmDJ-1 proteins were aligned with that of AtDJ-1D (AT3G02720) and OsDJ-1C (LOC_Os04g57590.1) and only DJ-1/PfpI domain of *H*. *Sapiens* DJ-1 (1PDV:A), *D*. *Melanogaster* DJ-1α (4E08:A), *S*. *cerevisiae* Hsp31 (4QYX:A) *C*. *albicans* Hsp31 (4LRU:A), *E*. *coli* Hsp31 (1PV2:A). The sequences were alignment by Clustal omega and alignment Fig. was generated by jalveiw. The conserved catalytic triad residues of for both DJ-1 and Hsp31 proteins are marked by filled triangle and star, respectively.

All GmDJ-1 proteins were found to have two DJ-1/PfpI domains (Fig. S2) like previously reported plant DJ-1 proteins from rice and *Arabidopsis*^20^. However, DJ-1 proteins from human, *Drosophila* and Hsp31 proteins from *E*. *coli*, yeast, and *C*. *albicans* were found to have only one DJ-1/PfpI domain. Thus, the N- and C- terminal DJ-1/PfpI domain of all GmDJ-1 proteins were aligned separately with that of other reported GLY III proteins (Figs 3, S3). DJ-1 proteins have a unique catalytic triad, consists of glutamate, cysteine and histidine residues^1^. Among these, glutamate and cysteine residues are found to be highly consistent in the N-terminal domains of GmDJ-1 proteins (Fig. 3). But, the position of the third catalytic residue (His) of this triad was found to be variable. GLY III protein of Hsp31 classes has His residue besides the conserved Cys (marked with a star), whereas DJ-1 proteins have His at a distal site from Cys (marked with a triangle). Among seven GmDJ-1 proteins, N-terminal of GmDJ-1C1 showed the absence of catalytically indispensable conserved Cys residue and thus, might lack GLY III activity. Among others, the N-terminal domain of GmDJ-1D1, GmDJ-1D2, and GmDJ-1D3 showed the catalytic triad similar to Hsp31 proteins; whereas GmDJ-1C2, GmDJ-1B and GmDJ-1A showed similarity with DJ-1 proteins. Moreover, the third conserved His residue of the catalytic triad could be replaced by either Tyr or Phe that has been found in different fungal and *Bombyx mori* DJ-1 proteins^1^. The almost similar pattern of sequence conservance was observed in the C-terminal domain of six GmDJ-1 proteins (except GmDJ-1C1) with that of other reported GLY III proteins (Fig. S3). GmDJ-1D3 showed the absence of evolutionarily conserved cysteine residue in the C-terminal domain, whereas other members showed similar preference towards Hsp31 or DJ-1 proteins like N-terminal domain.

### *GmDJ-1* transcripts showed diverse level of expression at various developmental stages

**Figure 4:**
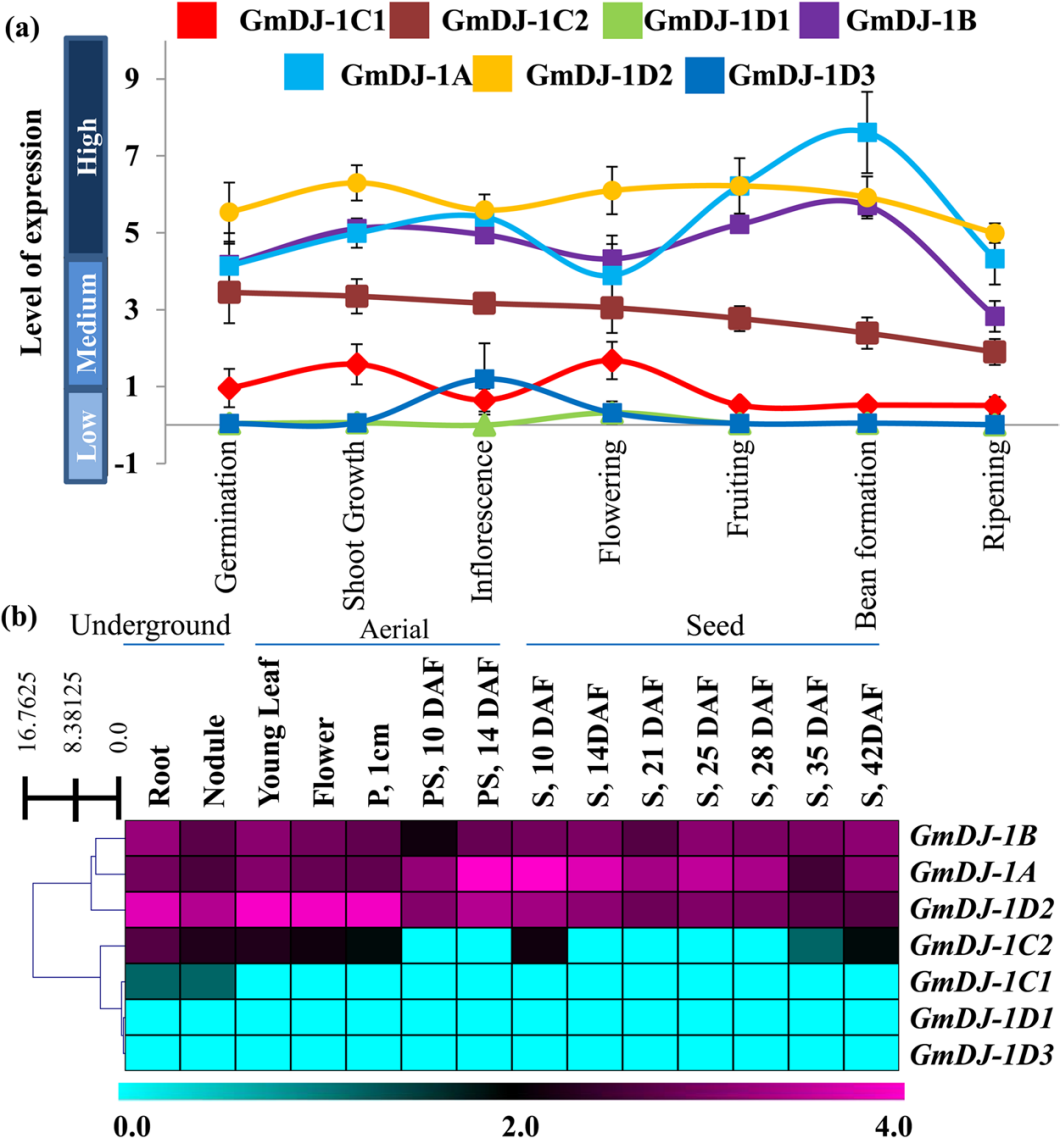
Expression profiling of *GmDJ-1* genes at different tissues and developmental stages. (**a**) Transcriptome data of *GmDJ-1* genes at different developmental stages including germination, main shoot growth, inflorescence development, flowering, fruiting, bean formation and final ripening were obtained from genevestigator (https://genevestigator.com/gv/doc/intro_plant.jsp). (**b**) RNA-Seq expression data of fourteen soybean tissues, such as Root, Nodule, Young Leave, Flower, Pod one cm, Pode Shell (10 day after flowering, DAF and 14 DAF), Seed (10 DAF,14 DAF, 21 DAF, 25 DAF, 28 DAF, 35 DAF, and 42 DAF) was retrieved from soybase database (http://soybase.org/soyseq/) and analyzed. Heat map with hierarchical clustering was performed using MeV software package. The color scale below the heat map indicates expression values; cyan color indicates low transcript abundance while purple indicates a high level of transcript abundance.

Plant development is a highly complex process that altered the expression of several genes drastically to meet the physiological and metabolic demand. To analyze the developmental regulation of *GmDJ-1* transcripts, expression of *GmDJ-1* genes was checked at different developmental stages. Soybean has seven distinct developmental stages- germination, main shoot growth, inflorescence formation, flowering, fruit formation, bean development and final ripening. Expression of all these *GmDJ-I* genes was investigated using genevestigator (https://genevestigator.com/gv/doc/intro_plant.jsp) based mRNA-seq data. Among the total of 7 *GmDJ-I* genes; *GmDJ-1*D2 showed the highest level of expression at all the developmental stages of soybean, except bean formation stage where *GmDJ-1*A showed the maximum expression (Fig. 4a). Overall, the analysis revealed that *GmDJ-1*B, *GmDJ-1*A and *GmDJ-1*D2 maintained high transcript abundance at all the developmental stages, followed by a medium level of expression of *GmDJ-1*C2, and the rest three members such as *GmDJ-1*C1, *GmDJ-1*D1, and *GmDJ-1*D3 maintained the low level of expression (Fig. 4a). Although no specific developmental regulation was observed for *GmDJ-1* genes, a contrasting level of expression was observed among seven *GmDJ-1* family members.

### Tissue-specific alteration of *GmDJ-1* transcripts

Previous studies observed a variable pattern of expression among different members of a gene family in different tissues^35–37^. Expression of *GmDJ-1* transcripts were analysed on fourteen different *Glycine max* tissues such as root, nodule, young leaf, flower, pod (One cm), pod-shell (PS; 10 day after fertilization (DAF) and 14 DAF), seed (S; 10 DAF, 14 DAF, 21 DAF, 25 DAF, 28 DAF and 35 DAF) that could be broadly divided into three part; underground, aerial and seed (Table S3). Two distinctive patterns of expression were observed among various *GmDJ-1* members (Fig. 4b). Expression of two members *GmDJ-1*D1 and *GmDJ-1*D3 were found to be lowest in all tissues, whereas *GmDJ-1*B, *GmDJ-1*A, and *GmDJ-1*D2 showed a high level of expression in all tissues (Fig. 4b) similar to developmental data. Expression of *GmDJ-1*C1 was found to be confined only to the underground tissue (root, nodule). However, in addition to a medium to high level of underground tissue-specific expression, *GmDJ-1*C2 showed significant expression at early stages of aerial tissues and mature seeds (Fig. 4b).

### Expression of *GmDJ-1* transcripts altered in response to environmental stimuli

**Figure 5:**
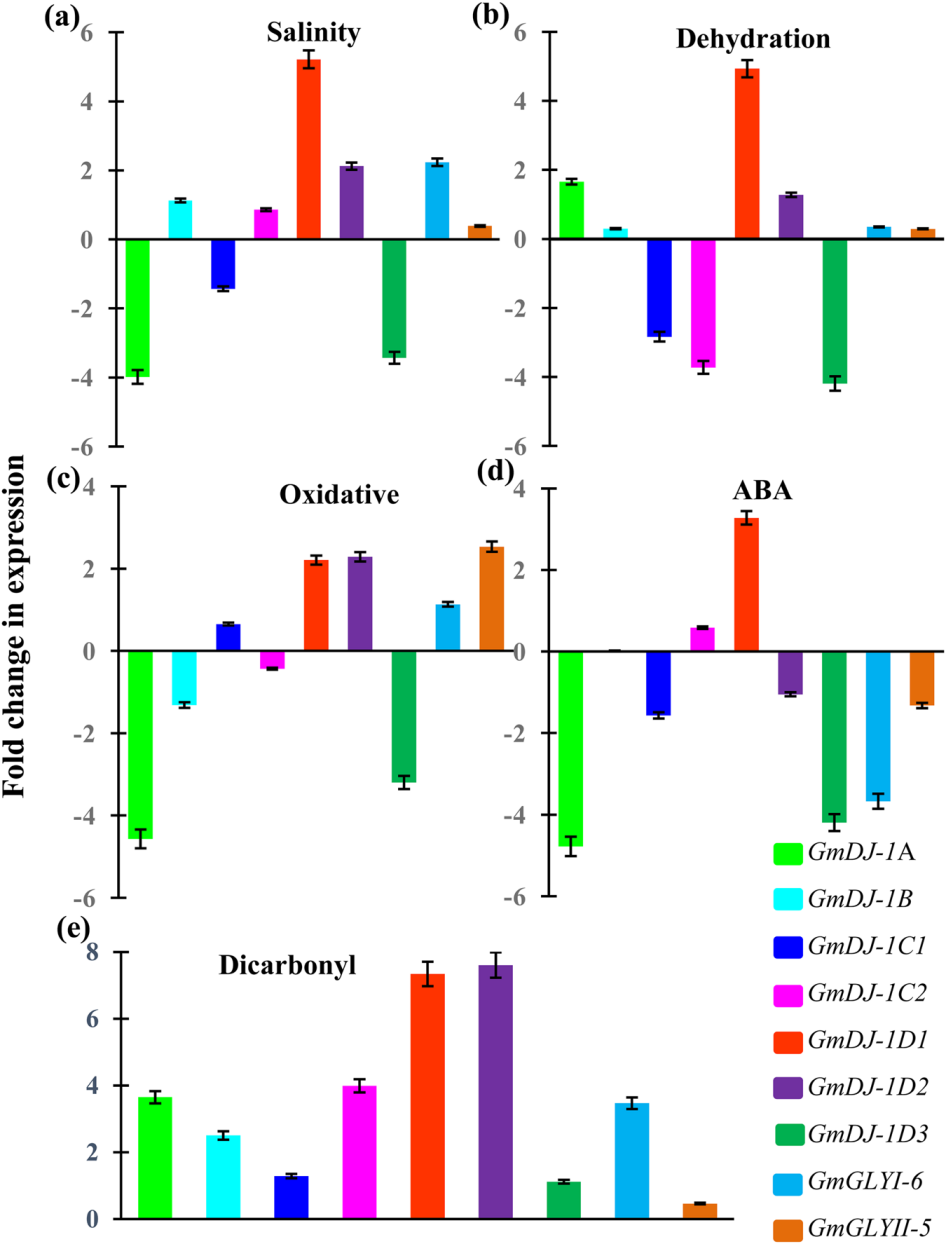
Expression analyses of *GmDJ-1* genes under different stress conditions. Expression of seven *GmDJ-1* genes along with two members from conventional GLY pathway (*GmGLYI*6 and *GmGLYII*5) was analyzed in response to various unfavorable conditions by qRT-PCR. Bar graphs showed the fold change in expression of *GmDJ-1* transcripts against salinity (**a**), dehydration (**b**), oxidative stress, H_2_O_2_ (**c**), ABA treatment (**d**) and dicarbonyl stress, exogenous MG (**e**). Expression analysis was performed in 15 days old soybean seedlings subjected to 8 hrs stress treatment by qRT-PCR. Error bars showed the standard deviation of three replicates.

To check the effect of environmental clues on the expression of *GmDJ-1* genes, a qRT-PCR based transcript analysis was performed in response to two abiotic stresses (salinity and dehydration), oxidative stress (H_2_O_2_), hormonal treatment (abscisic acid, ABA), and dicarbonyl stress (exogenous MG). Relative fold change in expression of *GmDJ-1* genes was analyzed using *Tubulin* as house-keeping gene; and *GmGLYI*−6 and *GmGLYII*−5 as positive control from the previous study^38^. Expression of *GmGLYI*−6 and *GmGLYII*−5 was found to be upregulated in response to salinity and dehydration, and downregulated under ABA treatment; that is quite similar to the previous report^38^. All members of the GmDJ-1 family showed an interesting pattern of up-regulation in response to exogenous MG stress (Fig. 5e). As MG is the direct substrate for GLY III enzymes and very toxic in nature, plants might try to neutralize them by upregulating the detoxifying genes. Expression of *GmDJ-1*D1 and *GmDJ-1*D2 genes have been found to be up-regulated in response other stresses (Fig. 5a–d). Transcript of *GmDJ-1*D1 showed two to seven folds up-regulation under all conditions; while *GmDJ-1*D2 showed one to eight folds upregulation, except ABA treatment with slide downregulation (Fig. 5). Moreover, *GmDJ-1*D3 was found to be the most down-regulated member of *GmDJ-1* family, followed by *GmDJ-1*A and *GmDJ-1*C1 (Fig. 5). However, other two members (*GmDJ-1*B and *GmDJ-1*C2) showed minor alteration in their transcript level to a very narrow range. This indicates the critical role of these members in the stress adaptation and modulation mechanisms of soybean.

### GLY III enzyme activity for GmDJ-1D2

**Figure 6:**
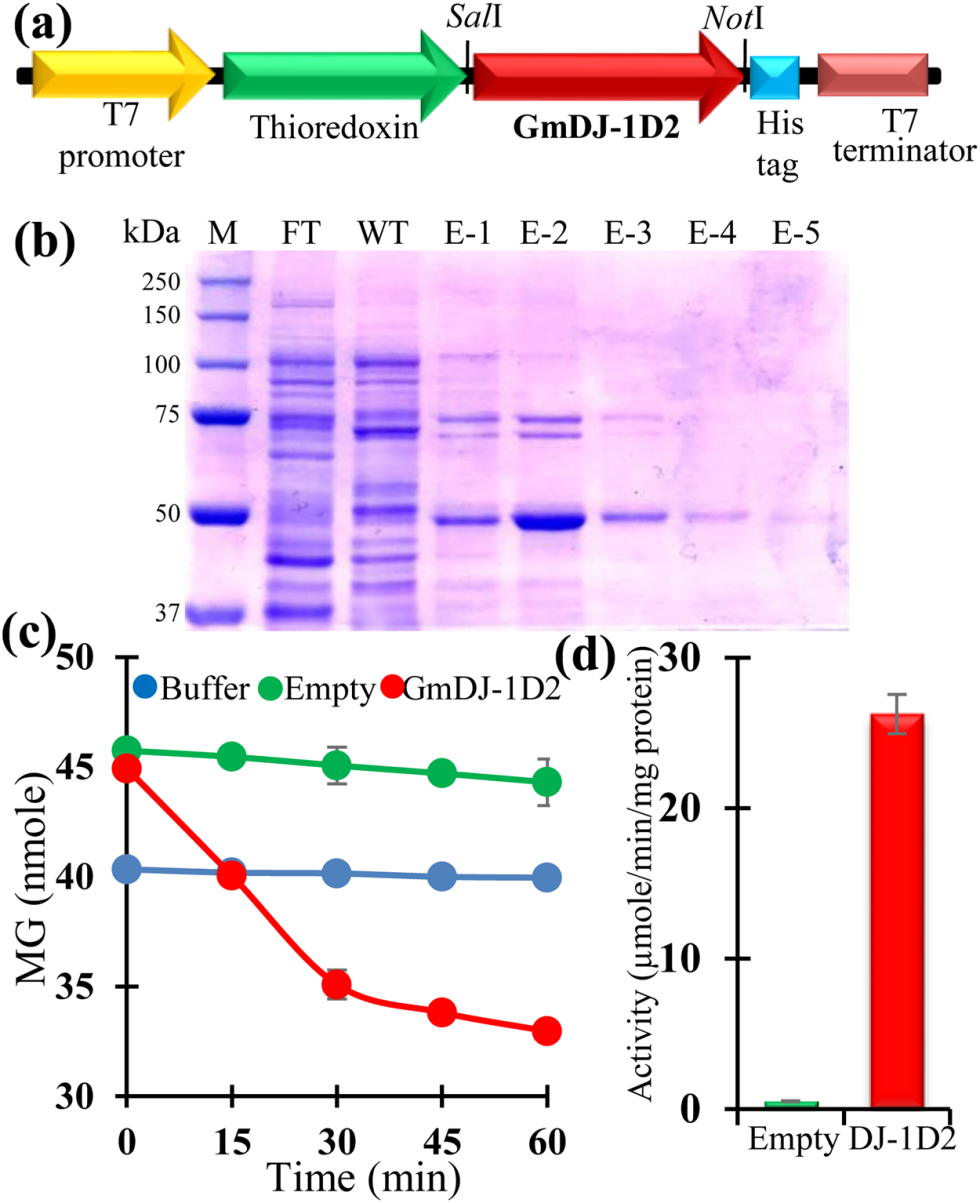
GLY III activity of the recombinant GmDJ-1D2 protein. Recombinant GmDJ-1D2 protein was tested for the functional GLY III enzyme activity. For that, GmDJ-1D2 was cloned into the SalI and NotI site of the pET201 vector to make the construct (**a**) used for protein expression. The recombinant protein was purified from *E*. *coli* BL21 (DE3) Rosetta cells and run into 12% SDS-PAGE gel, where FT: flow through, WT: wash through (40 mM imidazole), E1–5: (elution fractions using 200 mM imidazole). (**c**) MG depletion assay has been carried out in three different conditions for 60 min; (i) Only MG + buffer (blue circle), (ii) MG + buffer + empty vector extract (green circle) and (iii) MG + buffer + recombinant GmDJ-1D2 protein (red circle). This experiment clearly indicates the presence of functional GLY III enzyme activity for GmDJ-1D2 protein. (**d**) Specific GLY III activity of GmDJ-1D2 protein along with empty vector purified (Thioredoxin) was determined and presented as a bar diagram. Experiments were performed in triplicate and presented as mean ± standard deviation.

To check the GLY III activity of GmDJ-1 proteins, developmentally constitutively expressive (Fig. 4) and one of the highly MG-responsive members (Fig. 5e), *GmDJ-1*D2 was cloned in a bacterial expression vector pET201 (Fig. 6a). The recombinant Thioredoxin-GmDJ-1D2-His-tag protein was purified using Ni-NTA based affinity chromatography and the purified protein showed a prominent band of ~50 kDa (Fig. 6b). GLY III activity has been assayed in three different conditions; (1) Only MG was added (as buffer control), (2) MG was mixed with Ni-NTA purified Thioredoxin-His-tag protein from empty vector transformed cells (as empty vector control), and finally (3) MG and Ni-NTA purified recombinant GmDJ-1D2 protein was mixed (as main GmDJ-1D2 reaction). Both buffer and empty vector controls showed very insignificant change to the level of total MG in a reaction time of 60 min, while GmDJ-1D2 protein showed a significant and static reduction of MG over time (Fig. 6c). That indicates the ability of the GmDJ-1D2 protein to utilize MG as the substrate and acts as functional GLY III enzyme. This observation validates the functional role of GmDJ-1D2 protein as GLY III enzyme. The rate of MG utilization for GmDJ-1D2 was 26,234 µmole/min/mg protein in the present experimental condition, while that was only 0,502 µmole/min/mg protein for empty vector considering the linear range of MG depletion for GmDJ-1D2 (0 to 30 min) (Fig. 6d). The catalytic rate of GmDJ-1D2 is significantly higher as compared to AtDJ-1D (8,60 μmol/min/mg)^19^, lower than OsDJ-1C (58 μmol/min/mg)^20^.

### Various stress-responsive cis-acting regulatory elements are present in the promoters of *GmDJ*−1 genes

**Figure 7:**
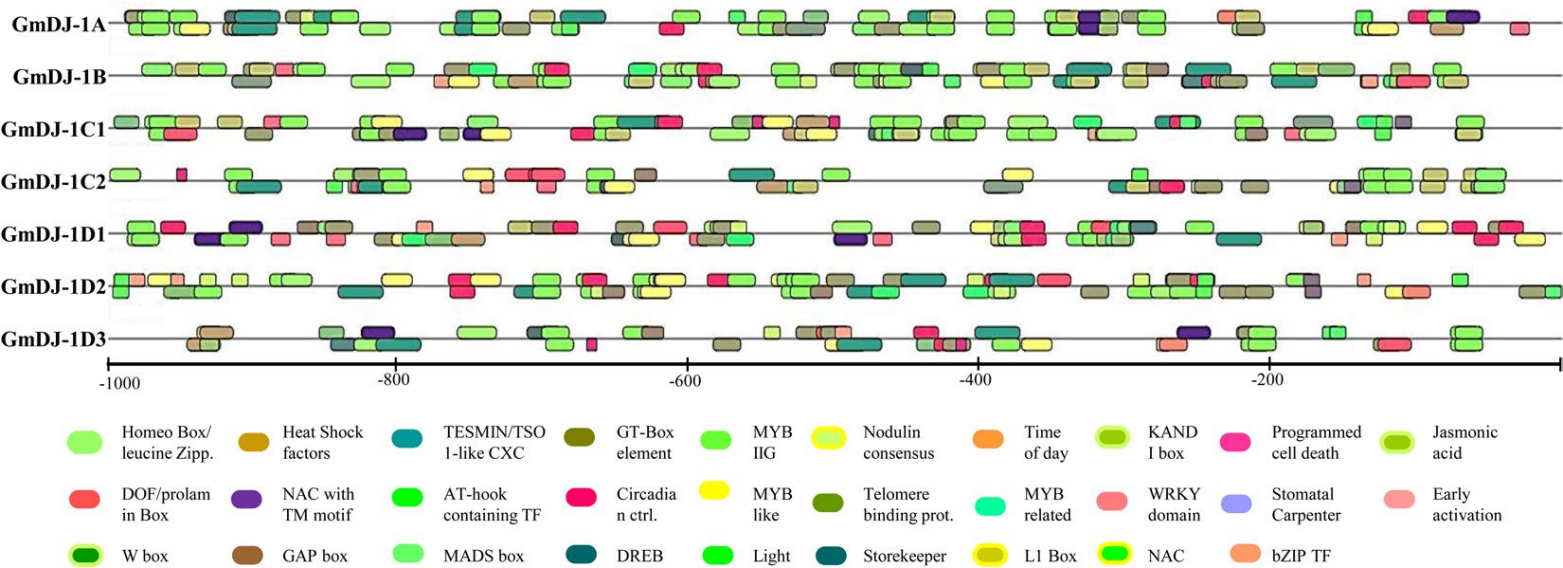
Analysis of *GmDJ-1* promoter sequences. The 1 kb upstream promoter sequence of transcription start site was retrieved and analyzed for the presence of various cis-regulatory elements. Different elements were identified and represented with different artworks. Both strands of DNA was presented in the figure, where the artworks up to the black line indicate positive strand and motifs down to the line indicates negative strand.

To investigate the cellular mechanism behind the altered expression of *GmDJ*−1 genes in response to various developmental stages and stresses, the 1 kb upstream region of the genes from transcription start site was analyzed *in silico* to identify the presence of cis-regulatory elements. This analysis identified the presence of several stress-related and hormone inducible motifs such as Homeo Box/ leucine Zipper, Heat Shock factors, CRC domain containing tesmin/TSO1-like CXC (TCX) factors, AT-hook containing transcription factors, Circadian control factors, DNA binding with one finger (DOF), Dehydration responsive element binding factors (DREB), GAP-Box (light response elements), Plant G-box/C-box bZIP proteins, GT-box elements, Jasmonate response element, Transcription repressor KANADI, L1 box (motif for L1 layer-specific expression), Light responsive element motif, MADS box proteins, MYB IIG-type binding sites, MYB-like proteins, MYB proteins with single DNA binding repeat, Plant specific NAC transcription factor binding site, Nodulin consensus sequence 1, NAC factors with transmembrane motif binding, Factors involved in programmed cell death response, Stomatal Carpenter, Sweet potato DNA-binding factor with two WRKY-domains, Storekeeper like transcriptional regulators, Telo box (plant interstitial telomere motifs), Time-of-day-specific cis regulatory elements, target of early activation tagged factors, W Box family found to be present in the promoter of at least 4 genes out of seven (Fig. 7). All these cis-acting regulatory elements play an important role to modulate the molecular switches of dynamic transcriptional regulation in response to developmental processes, stress responses, and hormonal signalling^39^. These motifs were found to be distributed randomly in both positive (top of the line) and negative strand (down to the line) of the promoters of *GmDJ*−1 genes (Table S5). The minimum number of 152 conserved motif binding sites were present on the promoter of *GmDJ*−1D3, while maximum 222 binding sites were found to be present on the *OsDJ*−1B promoter (Fig. 7). Among others, promoter of GmDJ-1C1, GmDJ-1A, GmDJ-1D1, GmDJ-1D2, and GmDJ-1C2 has 220, 204, 193, 191 and 170 conserved binding sites; respectively. Among the total 29 elements, homeobox-protein binding site matched 285 times, while time-of-day-specific cis-regulatory elements found to be matched 5 times; for all seven promoters.

## Discussion

Soybean (*Glycine max*) is one of the major vegetable protein and oil-producing legume plant, under serious threat due to various environmental stresses including drought, salinity, and osmotic stress^40,41^. Water deficit or impurity could shorten the flowering and seed-filling periods of soybean, and thus accelerate senescence and reduce productivity^42,43^. Available soybean genome database prompts us to identify novel stress modulating genes^44^. The current study focuses on the identification and expression analysis of unique DJ-1/PfpI domain-containing glyoxalase III (GLY III) proteins in *Glycine max*. Genome-wide analysis of soybean reveals the presence of eleven DJ-1 proteins encoded by seven genes (Table 1). Although most of the soybean genes expanded in a species-specific manner due to two gene duplication events of soybean occurred after the monocot/dicot split^31^, the number of *DJ-1* genes are found to be almost similar with that of monocot rice (six DJ-1 genes code for eleven proteins), and dicot *Arabidopsis* (six DJ-1 genes code for twelve proteins) and *Medicago* (five DJ-1 genes code for six proteins). However, the number of conventional glyoxalase genes is 2.2 to 4 times more in soybean as compared to *Arabidopsis* and rice^38^. Different GmDJ-1 proteins were found to be localized in various organelles, such as mitochondria, cytosol, and chloroplasts (Table 1). Subcellular localization of three AtDJ-1 family members; AtDJ-1a, AtDJ-1b, and AtDJ-1c were analyzed experimentally in transgenic *Arabidopsis* plants^28^. Among them, AtDJ-1b and AtDJ-1c were found to be targeted in plastids, while AtDJ-1a shown cytosolic localization. Recently, stress-responsive translocation potential of Hsp31 protein from cytosol to mitochondria has been reported in yeast^17^.

Like other plant DJ-1 proteins, all GmDJ-1 proteins have two repeated DJ-1/PfpI domain (except GmDJ-1C1.2 and GmDJ-1C1.3) connected by a linker sequence (Fig. S2). The average size of the DJ-1/PfpI domain of soybean has been found to be ~165 amino acids (Fig. S2). However, the average size of rice and *Arabidopsis* DJ-1/PfpI domain were reported to be around 140 to 150 amino acids, while *E*. *coli* has a domain size of 170 amino acids^20^. Moreover, the size of DJ-1 domain of Hsp31 proteins from *E*. *coli*, yeast, and *C*. *albicans* was observed more than 200 amino acids (Fig. 3). The precise correlation between domain size and GLY III enzyme activity could not observe. Currently, it is clear that Hsp31 proteins of prokaryotes and lower eukaryotes have a relatively longer length of DJ-1/PfpI domain as compared to that of higher organism DJ-1 proteins. To investigate the difference between domain size and evolutionary history of DJ-1 and Hsp31 proteins, a comprehensive phylogenetic analysis was performed among enzymatically active GLY III proteins from a wide range of taxonomically diverse species (Fig. 2). This study revealed significant information regarding the evolution of GLY III proteins. All Hsp31 proteins of *E*. *coli*, yeast, *C*. *albicans* and *S*. *pombe* are completely distinct from the evolutionary clade of DJ-1 proteins. Interestingly, one of the fungal species, *S*. *pombe* is the only organism till date has been reported with both DJ-1 and Hsp31 class of proteins with active GLY III enzyme^1^. *S*. *pombe* DJ-1 protein forms cluster with DJ-1 proteins of other organisms, while Hsp members form a cluster with Hsp counterparts (Fig. 2). Hsp31 proteins were also found to be distributed widely among different other fungal species^1^, indicating the retention of Hsp31 proteins during fungal evolution. The presence of both DJ-1 and Hsp31 proteins in fungus appears to be an important junction of evolution. In terms of the metazoans divergence from fungal lineages, DJ-1 proteins appeared before this and Hsp31 proteins lost after that^1^. This phenomenon needs to be investigated further to confirm the evolutionary appearance DJ-1 proteins with simultaneous diminishing Hsp31 proteins.

It has been reported earlier that DJ-1/PfpI domain-containing proteins played important role in the scavenging of reactive species produced under oxidative stress in the animal system^5^. Expression of DJ-1 transcripts has been found to be altered in response to various external factors in rice^20^. A significant up-regulation of all *OsDJ-1* transcripts were observed in response to external dicarbonyl stress (MG stress). Apart from that, expression of *OsDJ-1* transcript was up-regulated in response to various other stresses including salinity, drought, cold, heat and oxidative stress^20^. Similarly, expression of *AtDJ-1A* transcript was found to be up-regulated in response to various external factors including strong light, CuSO_4_, H_2_O_2_ and Methyl viologen^28^. Recently, overexpression of a DJ-1 homolog, yeast Hsp31, in model plant *Nicotiana tabacum* plants showed dual biotic and abiotic stress tolerance^45^. In the present study, expression of the *GmDJ-1*D1 transcript was found to be the most upregulated member in response to various abiotic stresses- salinity and dehydration, oxidative stress, ABA treatment and dicarbonyl stress (Fig. 5). Significantly, all *GmDJ-1* members *showed* up-regulation in response to exogenous MG stress (Fig. 5e), along with conventional GLY pathway (*GmGLYI*6 and *GmGLYII*5). The significant increase in the level of MG in response to stress^46^, might induce the expression of MG metabolizing enzymes as substrate inducible mechanism. A similar level of substrate (MG) induced upregulation was overserved for *OsGLYI*11.2^47^ and some other metabolic enzymes^48^. Thus, a differential pattern was observed with two different sets of genes for two different roles. One set of DJ-1 genes might involve in the developmental/tissue-specific signals; while another set is assigned for the stress-specific modulation. This would be interesting to analyze the expression and role of these genes in different physiological needs.

## Materials and Methods

### Identification and *in silico* characterization of homologous *DJ-1* genes in soybean

All putative DJ-1 proteins in soybean genome were identified by using previously characterized *Arabidopsis* DJ-1 member with maximum GLY III activity, AtDJ-1D protein sequence (AT3G02720/ Q9M8R4) as a query in the BLASTP search of soybean genome database (Wm82.a2.v1) (http://www.soybase.org/) with an e-value of 1.0^49^ and identified eleven members. Each of these newly identified members was subsequently used as a secondary query, but no new members appear. All these output protein sequences were analyzed individually for the presence of DJ-1/PfpI domain (PF01965.19) using Pfam (http://www.sanger.ac.uk/Software/Pfam) with an e-value of 1.0. All the identified putative DJ-1 proteins of soybean were nomenclature as prefix “Gm” for *Glycine max*, followed by DJ-1 and English letter (A-D) depending on their orthologous member at *Arabidopsis* as mentioned previously^30^. Multiple genes matching having same orthologous member were named by adding a hyphen ‘-’ followed by Arabic numbers after them and alternate splice forms were represented by adding Arabic numbers after “.” sign sequentially. The chromosomal location of all the putative *GmDJ-1* genes was identified from the soybase browser (http://soybase.org/gb2/gbrowse/gmax1.01/)^49^ to draw the chromosomal map. Gene duplication was analyzed using plant genome duplication database (http://chibba.agtec.uga.edu/duplication/index/downloads)^50^ for soybean. Divergence time (in millions of years) was calculated for each gene pair considering a rate of 6.1 × 10^−9^ substitutions per site per year^31^. Thus, divergence time (T) = Ks/(2 × 6.1 × 10^−9^)X10^−6^ Mya.

Different physio-chemical properties of the identified proteins including molecular weight and isoelectric point were calculated using Prot-Param software tool (http://web.expasy.org/protparam/) with default parameters. Localization of each GmDJ-1 proteins was predicted using default parameters of CELLO v.2.5: sub-cellular localization predictor (http://cello.life.nctu.edu.tw/)^51^, pSORT prediction software (http://wolfpsort.org/)^52^ and ChloroP (http://www.cbs.dtu.dk/services/ChloroP/)^53^.

### Multiple sequence alignment and phylogenetic analysis

To investigate the phylogenetic relationships among DJ-1 proteins from various species, DJ-1 sequence were retrieved from different databases such as NCBI (http://www.ncbi.nlm.nih.gov/), PDB (http://www.rcsb.org/pdb/home/home.do), RGAP7 (http://rice.plantbiology.msu.edu/), TAIR (https://www.arabidopsis.org/) and soybase (http://www.soybase.org/) (Text S2). Multiple sequence alignment was performed using Clustal Omega (https://www.ebi.ac.uk/Tools/msa/clustalo/) with default parameters^54^ and the best tree model was selected using ProtTest 2.4 server with akaike information criterion (AIC) (http://darwin.uvigo.es/software/prottest2_server.html)^55^. To identify the conserved active site residues the alignment was edited by Jalview^56^. The phylogenetic tree was constructed using the Maximum Likelihood method based on the Whelan And Goldman (+) Freq. model with gamma-distributed invariant sites (G + I) distribution^57^ of MEGA 7.0^58^ with 500 bootstrap replicates. Initial tree(s) for the heuristic search were obtained automatically by applying Neighbor-Join and BioNJ algorithms to a matrix of pairwise distances estimated using a JTT model, and then selecting the topology with superior log-likelihood value.

### Expression analysis of *GmDJ-1* genes at different developmental stages

Expression patterns of *GmDJ-1* genes at different developmental stages were determined using the publically available transcriptomes data from genevestigator database (https://genevestigator.com/gv/doc/intro_plant.jsp). Seven different developmental stages of soybean are Germination, Main Shoot Growth, Inflorescence formation, Flowering, Fruiting Bean development, and final ripening. Corresponding mean expression data was downloaded with standard deviation and scatter diagram was generated.

### Expression analysis using RNA-Seq Atlas of *Glycine max*

To analyze the tissue-specific expression data of seven *GmDJ-1* genes, their corresponding probe sets were identified using the soybase tool (http://www.soybase.org/correspondence/index.php). Normalized transcript data was downloaded from soybase (http://soybase.org/soyseq/) for fourteen different soybean tissues including root, nodule (underground tissues); leaf, flower, pod-shell 10 day after flowering (DAF), pod-shell 14 DAF, one cm pod (aerial tissues); and different stages of seed development (seed of 10 DAF, 14 DAF, 21 DAF, 25 DAF, 28 DAF, 35 DAF and 42 DAF). This normalized expression (log_10_ transformed) was used to generate heatmap and hierarchical clustering using the Institute for Genomic Research MeV software package^59^.

### Plant material, stress treatments, and qRT-PCR

To analyze the expression of *GmDJ-1* genes using RT-PCR, fifteen days old soybean (*Glycine max L*. variety Sohag) seedlings were treated with either normal water or 200 mM NaCl or 0.1 mM H O or 0.01 mM MG or 10 mM ABA solution for depicting experimental control, or salinity, or oxidative, or dicarbonyl or hormonal stress, respectively. To mimic dehydration stress, seedlings were placed on filter paper. After 8 h, shoot tissues were collected from all these seedlings (with triplicates) and total RNA was extracted using TRIzol® Reagent (Thermo Fisher Scientific, USA). First-strand cDNA was synthesized using RevertAid First Strand cDNA Synthesis Kit (Thermo Fisher Scientific, USA). Gene-specific primer for seven *GmDJ-1* genes was designed using Primer-Blast (http://www.ncbi.nlm.nih.gov/tools/primer-blast/), and primer for house-keeping soybean *Tubulin* gene was taken from literature^60^. All these primers were synthesized from Macrogen (http://dna.macrogen.com/eng/) and listed in Table S4. The qRT-PCR was conducted in triplicate according to the previously described protocol^61^ and the fold change in expression was calculated using the 2^(-Delta Delta Ct) method^62^.

### Expression and purification of recombinant GmDJ-1D2 protein, and GLY III enzyme activity

Complementary DNA sequence of GmDJ-1D2 was synthesized from GenScript (https://www.genscript.com/) and subsequently cloned into the SalI and NotI sites of the pET201 vector using gene-specific primers (DJ-1D2_Sal1_FOR: 5′ gggagagtcgagATGGCTCCGAAGAAGGTTC 3′ and DJ-1D2_NotI_REV: 5′ gggagatgcggccgcAGATACTTGAATACCAAG3′) specific amplification. The positive plasmids were confirmed by sequencing, transformed in *E*. *coli* BL21 (DE3) Rosetta cells. The expression was induced at 18 °C with 0.1 mM of IPTG for overnight and purified using Ni-NTA based affinity chromatography as mentioned previously^46^. The purified protein was quantified using the Bradford method^63^, and the purity was checked in SDS/PAGE. GLY III enzyme activity was performed using 1 mM MG as substrate (M0252, Merck, Germany) and recombinant protein (2–10 µg) in 20 mM potassium phosphate buffer (pH 7.0) for 60 min; and the remain MG at various steps was determined using the colorimetric 2,4-dinitrophenylhydrazine method as described previously^19^.

### Analysis of cis-regulatory elements on promoter

To identify the presence and number of transcription factor binding sites (TFBS), 1 kb 5′ upstream of all seven *GmDJ-1* genes was retrieved from the soybase database. The sequences were analyzed using MatInspector tool from the Genomatix software suite (http://www.genomatix.de/cgi-bin/matinspector_prof/mat_fam.pl?)^64^.

## Conclusions

Taken together, a detailed *in silico* genome-wide analysis of soybean unique glyoxalase III gene family (*GmDJ-1*) has been carried out. This is the first insight study of DJ-1 family from any legume plant. Soybean genome contains seven *GmDJ-1* genes that code for total eleven GmDJ-1 proteins. A detailed analysis of these members was carried out in terms of their structure, chromosomal location, sub-cellular localization and evolutionary relationship. Phylogenetic analyses of different characterized GLY III enzymes revealed the evolutionary divergence of Hsp31 and DJ-1proteins. Moreover, stress-specific expression data narrow down few promising stress-responsive members- *GmDJ-1*D1 and *GmDJ-1*D2 that could be used to generate stress tolerant soybean plant. The data presented here will serve as a mine for further functional characterization and validation of GLY III enzymes in soybean.

## Electronic supplementary material

Supplementary Information
