# Supplementary material for: Genome-wide dissection and expression profiling of unique glyoxalase III genes in soybean reveal the differential pattern of transcriptional regulation
Source: Sci Rep. 2018 Mar 19;8:4848. doi: 10.1038/s41598-018-23124-9 (PMC5859077; doi:10.1038/s41598-018-23124-9)
Supplement: Supplementary file 1 — Supplementary Information [file 41598_2018_23124_MOESM1_ESM.pdf]

Genome-wide dissection and expression profiling of unique glyoxalase III genes in soybean reveal the differential pattern of transcriptional regulation

Short title: Unique *glyoxalase III* genes in soybean

Tahmina Islam[1], Ajit Ghosh[2,3,*]

[1]Plant Breeding and Biotechnology Laboratory, Department of Botany, University of Dhaka, Dhaka-1000, Bangladesh; [2]Department of Biochemistry and Molecular Biology, Shahjalal University of Science and Technology, Sylhet-3114, Bangladesh; and [3]Department of Plant Developmental Biology, Max Planck Institute for Plant Breeding Research, Köln 50829, Germany.

Ajit Ghosh (E-mail: ajitghoshbd@gmail.com)
Tahmina Islam (E-mail: subarna.islam@gmail.com)

*To whom correspondence should be addressed:
Ajit Ghosh Ph.D.,
Department of Biochemistry and Molecular Biology,
Shahjalal University of Science and Technology,
Sylhet-3114, Bangladesh;
Phone: +880-821-713491, Ext. 409 (O); +88-01679436700 (M);
Fax: +880-821-715257.
E-mail: ajitghoshbd@gmail.com

Table S1 Divergence between *DJ-1* gene pairs in Soybean

LOCUS_1	LOCUS_2	Ka	Ks	Ka/Ks	divergence time (Mya)
Glyma.02G131600 (GmDJ-1C1)	Glyma.07G213200 (GmDJ-1C2)	0.055	0.1102	0.4991	9.0328
Glyma.11G207900 (GmDJ-1D1)	Glyma.18G045900 (GmDJ-1D2)	0.061	0.136	0.4485	11.1475
Glyma.12G228600 (GmDJ-1B)	Glyma.13G271200 (GmDJ-1A)	0.0256	0.1363	0.1878	11.1721

Table S2 Number of exon and introns in the all splice variants of *GmDJ-1* genes

Gene	No. of exons	No. of introns	No. of introns		
			CDS	5'UTR	3'UTR
GmDJ-1A	7	6	6	0	0
GmDJ-1B	7	6	6	0	0
GmDJ-1C1.1	6	5	5	0	0
GmDJ-1C1.2	6	5	5	0	0
GmDJ-1C1.3	5	4	4	0	0
GmDJ-1C2.1	7	6	6	0	0
GmDJ-1C2.2	7	6	6	0	0
GmDJ-1D1	8	7	7	0	0
GmDJ-1D2.1	5	4	4	0	0
GmDJ-1D2.2	4	3	3	0	0
GmDJ-1D3	5	4	4	0	0

CDS, protein-coding sequence; UTR, untranslated region

Table S3 Expression analysis of soybean *DJ-1* genes through RNA-seq data

**Gene**	**Root**	**Nodule**	**Young leaf**	**Flower**	**Pod 1 cm**	**PS 10 DAF**	**PS 14 DAF**	**Seed 10 DAF**	**Seed 14 DAF**	**Seed 21 DAF**	**Seed 25 DAF**	**Seed 28 DAF**	**Seed 35 DAF**	**Seed 42 DAF**
GmDJ-1C1	2	2	1	1	0	0	1	0	0	0	0	0	0	0
GmDJ-1C2	10	5	5	4	3	1	1	4	1	1	1	0	2	3
GmDJ-1D1	0	0	0	0	0	0	0	1	0	0	0	0	0	0
GmDJ-1B	25	11	21	15	12	4	13	15	17	10	21	17	17	22
GmDJ-1A	15	9	19	13	12	24	123	193	66	30	44	32	8	21
GmDJ-1D2	68	36	94	88	88	19	37	29	22	14	18	16	11	10
GmDJ-1D3	0	0	0	0	0	0	0	0	0	0	0	0	0	0

Table S4 List of primers used in the study

Sl. no	Primer name	Sequence (5'-3')	Product size
1	GmDJ-1C1_FOR	AGAAAAAGAAGGTTTCCAGAGGAC	212
	GmDJ-1C1_REV	AAGGACACTAGGGGTGTGGT	
2	GmDJ-1C2_FOR	CGAAGAAGGTCCTGGTTCCC	183
	GmDJ-1C2_REV	GATCGGAACACGCGGAAATG	
3	GmDJ-1D1_FOR	GCTGGCATTTGGAGGCAAAA	183
	GmDJ-1D1_REV	TGACCTGGCTTAGCAGTTGG	
4	GmDJ-1B_FOR	ATGGCATTGCGTCATTTACGA	120
	GmDJ-1B_REV	CGTGGTGGAAGAGAGTGAGG	
5	GmDJ-1A_FOR	TCTCCCCGGAGGTTTACAGG	135
	GmDJ-1A_REV	CAAGGCCCAAGCACTACCG	
6	GmDJ-1D2_FOR	CCTCCTGTTAAACCCGTGCT	135
	GmDJ-1D2_REV	GAAATGGCGAATGAGCTCCG	
7	GmDJ-1D3_FOR	TCGCAAATGCACGGGTTTTC	250
	GmDJ-1D3_REV	AAAAGGAACCTTGGCCTGGA	

Table S5 Detailed information about the identified cis-regulatory motifs

Matrix Family	p-value	Match Total	Common to #sequences	GmDJ-1A	GmDJ-1B	GmDJ-1C1	GmDJ-1C2	GmDJ-1D1	GmDJ-1D2	GmDJ-1D3	Detailed Family Information
P$AHBP	0,007563	285	7	58	51	44	31	27	47	27	Arabidopsis homeobox protein
P$AHLF	0,026885	61	7	12	14	9	6	10	9	1	AT-hook containing transcription factors
P$CCAF	0,017363	99	7	11	13	18	10	18	20	9	Circadian control factors
P$DOFF	0,013494	61	7	6	8	11	7	9	10	10	DNA binding with one finger (DOF)
P$DREB	0,975665	6	5	1	1	1	0	1	0	2	Dehydration responsive element binding factors
P$GAPB	0,235508	13	6	0	1	1	2	3	3	3	GAP-Box (light response elements)
P$GBOX	0,834344	11	5	1	1	3	0	0	1	5	Plant G-box/C-box bZIP proteins
P$GTBX	0,003889	123	7	19	21	20	15	18	15	15	GT-box elements
P$HEAT	0,037797	28	7	7	3	8	3	3	2	2	Heat shock factors
P$JARE	0,696344	14	5	0	2	4	0	2	3	3	Jasmonate response element
P$KAN1	0,029521	46	7	5	7	8	7	11	2	6	Transcription repressor KANADI
P$L1BX	0,015077	80	7	9	21	15	17	6	5	7	L1 box, motif for L1 layer-specific expression
P$LREM	0,044325	12	7	1	1	2	3	1	1	3	Light responsive element motif
P$MADS	0,015077	36	7	5	6	6	3	12	1	3	MADS box proteins
P$MIIG	0,005752	63	7	10	12	11	9	7	7	7	MYB IIG-type binding sites
P$MYBL	0,000993	77	7	8	13	9	11	14	17	5	MYB-like proteins
P$MYBS	0,005752	59	7	5	15	6	9	11	9	4	MYB proteins with single DNA binding repeat
P$NACF	0,02094	20	7	2	1	5	3	4	3	2	Plant specific NAC transcription factors
P$NCS1	0,041146	26	7	1	3	3	2	4	6	7	Nodulin consensus sequence 1
P$NTMF	0,032601	33	7	7	1	4	6	8	2	5	NAC factors with transmembrane motif
P$PCDR	0,769453	6	4	0	0	2	1	0	1	2	Factors involved in programmed cell death response
P$SCAP	0,687495	11	5	1	1	5	2	0	2	0	Stomatal Carpenter
P$SPF1	0,314792	14	6	2	1	3	2	5	1	0	Sweet potato DNA-binding factor with two WRKY-domains
P$STKL	0,039155	12	7	1	2	2	3	2	1	1	Storekeeper like transcriptional regulators
P$TCXF	0,045892	86	7	19	12	11	6	9	16	13	CRC domain containing tesmin/TSO1-like CXC (TCX) factors
P$TELO	0,72496	13	6	2	3	1	1	4	0	2	Telo box (plant interstitial telomere motifs)
P$TODS	0,855091	5	4	0	0	1	1	0	2	1	Time-of-day-specific cis regulatory elements
P$TOEF	0,041146	28	7	5	6	1	4	4	5	3	Target of early activation tagged factors
P$WBXF	0,996243	24	5	6	2	6	6	0	0	4	W Box family
Total:				204	222	220	170	193	191	152	

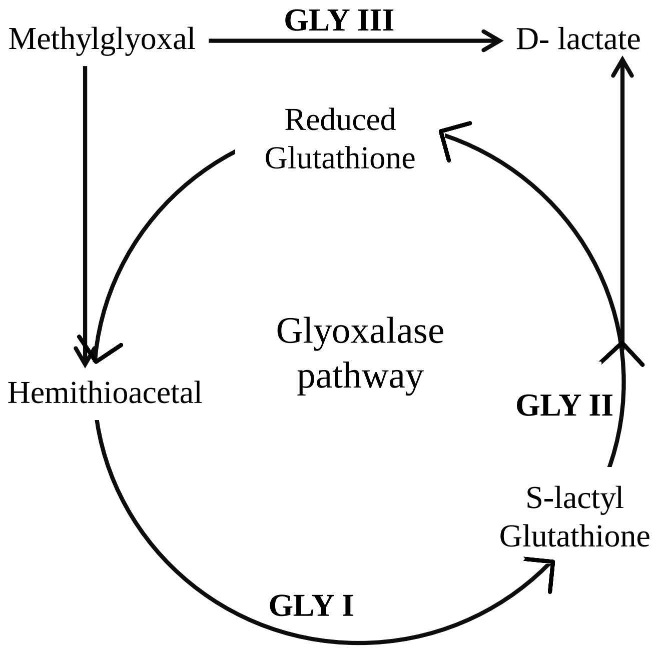

Figure S1 Conventional and alternative Glyoxalase pathway. Conventional Glyoxalase pathway has two enzymes, Glyoxalase I (GLYI) and Glyoxalase II (GLYII). GLYI acts on hemithioacetal a non-enzymatic adduct of Methylglyoxal and reduced glutathione, to form S-lactyl glutathione. GLYII converts S-lactyl glutathione to D-lactate and reduced glutathione. But the alternate unique glyoxalase pathway consists of one enzyme; Glyoxalase III (GLYIII) enzyme with DJ-1/PfpI domain that could do the conversion directly without the help of any cofactor.

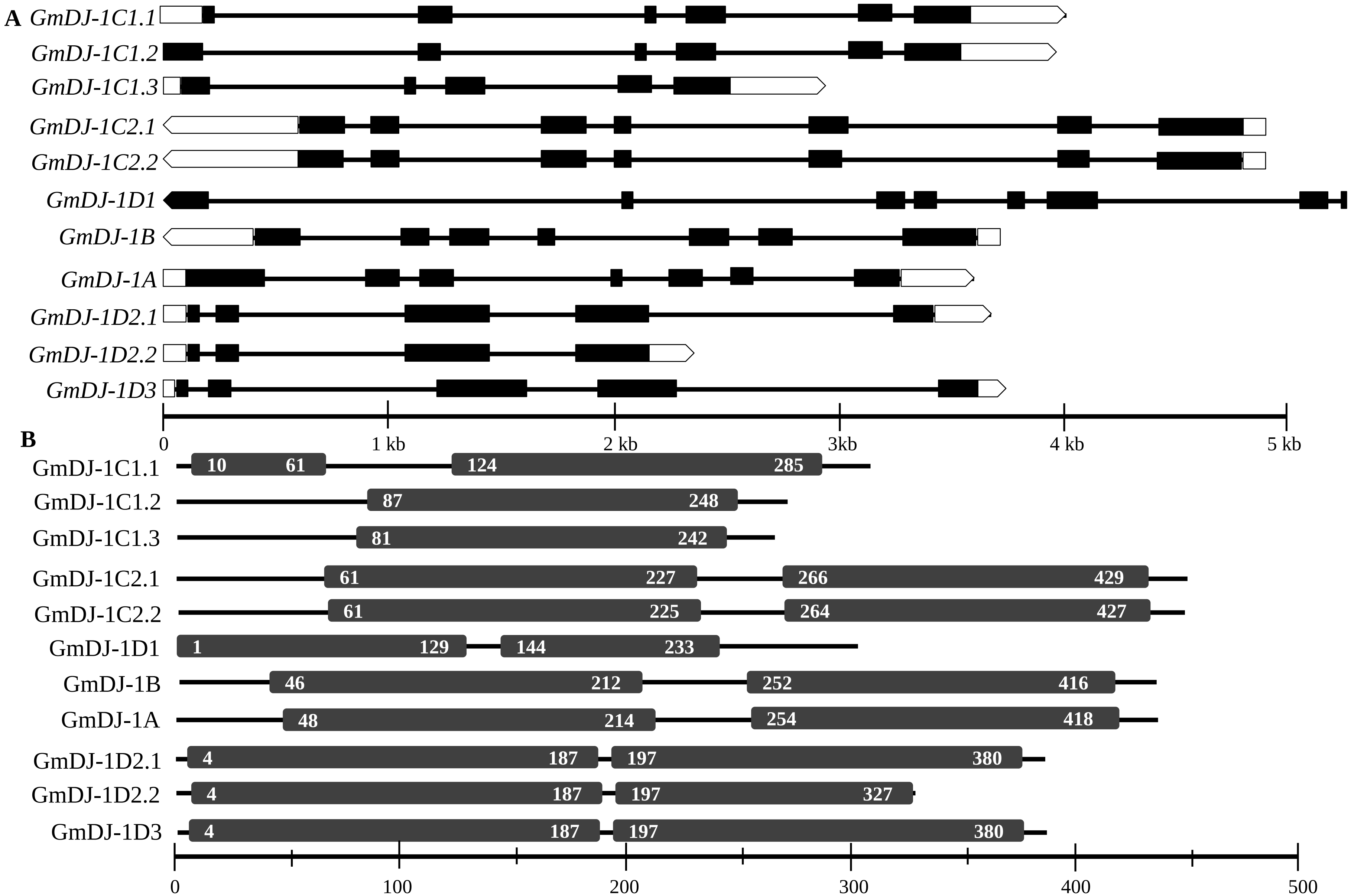

Figure S2 Exon-intron organization of *GmDJ-1* genes and Domain architecture of GmDJ-1 proteins. (A) Gene structure of all eleven GmDJ-1 transcripts was generated using soybase database information. Exons are shown by filled black boxes, introns are indicated by black lines and UTRs by empty boxes. The arrow of 3'UTR indicates the direction of the transcript. **(B)** All eleven GmDJ-1 proteins were analyzed by Pfam for the presence of DJ-1/PfpI domain. All GmDJ-1 proteins have two domains (represented by a black box), except GmDJ-1C-1.2 and GmDJ-1C-1.3. The length of the domain is indicated by an exact amino acid number inside the box. The relative scale of both transcripts and proteins are presented below each Fig.

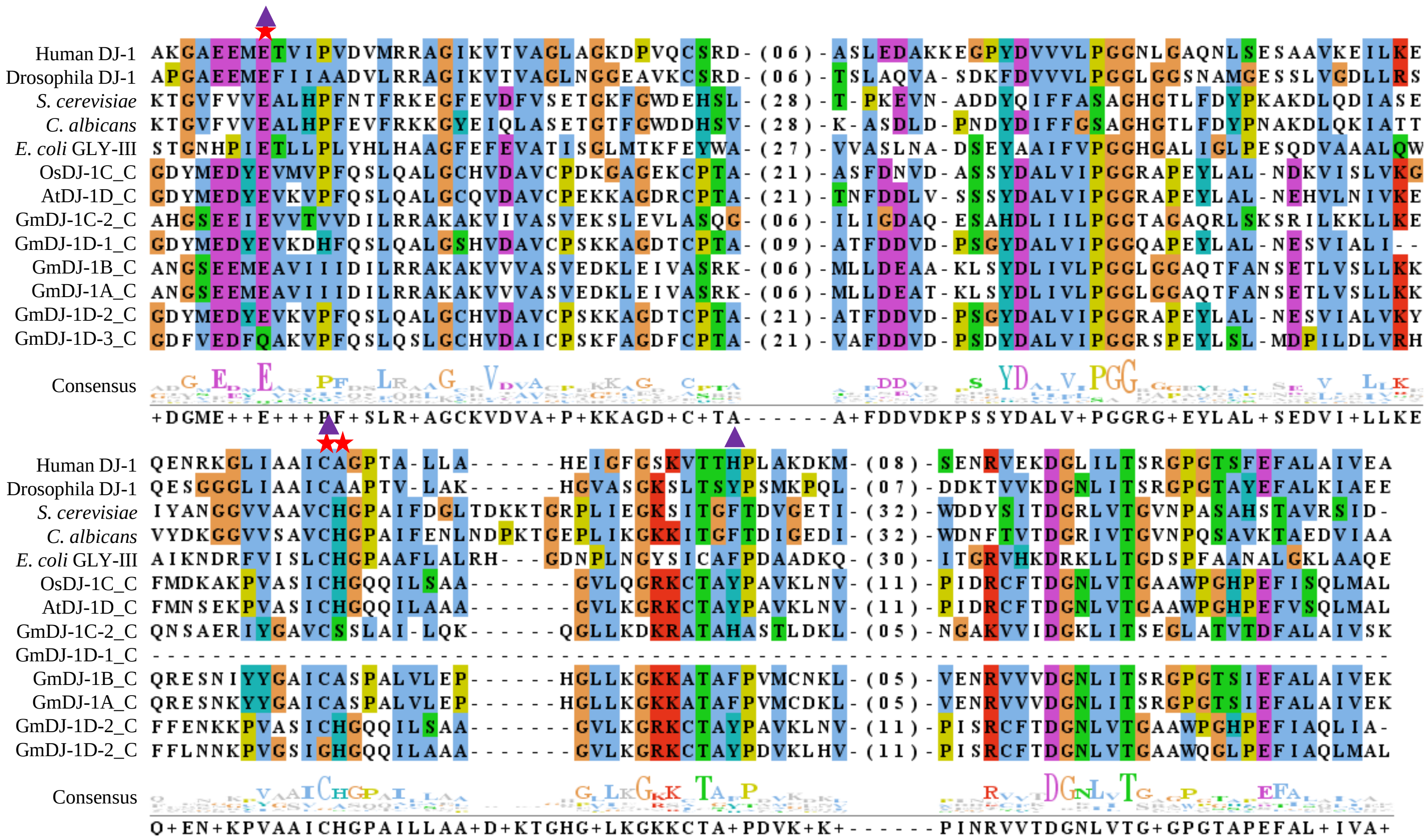

Figure S3 Multiple sequence alignment of C-terminal domain of GmDJ-1 proteins with other characterized GLYIII proteins from various species. Putative C-terminal DJ-1/PfpI domain of all six GmDJ-1 proteins (except GmDJ-1C-1) were aligned with that of AtDJ-1D (AT3G02720) and OsDJ-1C (LOC_Os04g57590.1) and only DJ-1/PfpI domain of *H. sapiens* DJ-1 (1PDV:A), *D. melanogaster* DJ-1α (4E08:A), *S. cerevisiae* Hsp31 (4QYX:A) *C. albicans* Hsp31 (4LRU:A), *E. coli* Hsp31 (1PV2:A). The sequences were alignment by Clustal omega and alignment figure was generated by jalveiw. The conserved catalytic triad residues of for both DJ-1 and Hsp31 proteins are marked by filled triangle and star, respectively.

S2 Text. Outcome of Prottest analysis

```
Title:     Phylogenetic sequence analysis
Submission date: 10/19/2016-15:51:22"
Check the automatically converted alignment!
[original alignment]
ProtTest icon


ProtTest - 2.4                          Selection of models of protein
evolution
(c) 2004                          Federico Abascal, Rafael Zardoya, David
Posada
(FA, DP)            Facultad de Biologia, Universidad de Vigo, 36200
Vigo, Spain
(RZ)                   Museo Nacional de Ciencias Naturales, 28006
Madrid, Spain
Contact:                                  fedeabascal@yahoo.es,
dposada@uvigo.es
--------------------------------------------------------------------------
-------

Wed Oct 19 16:51:29 CEST 2016
OS = Linux (2.6.34.10-0.6-desktop)


ProtTest options
----------------
  Alignment file........... :
/srv/www/vhosts/darwin.uvigo.es/serving/prottest/tmp/alignment_395
  Tree..................... : BioNJ
  StrategyMode............. : Fast (optimize branch lengths & model)
  Candidate models......... :
    Matrices............... : JTT LG DCMut MtREV MtMam MtArt Dayhoff WAG
RtREV CpREV Blosum62 VT HIVb HIVw
    Distributions.......... : +I +G +I+G
    Number of rate categ... : 4
    Observed frequencies... : true
  Statistical framework
    Sort models according to....: -lnL
    Sample size.................: 0.0 (not calculated yet)
      sampleSizeMode............: Total number of characters (aligment
length)
  Other options:
    Display best tree in ASCII..: false
    Display best tree in Newick.: true
    Verbose.....................: true


**********************************************************
  Reading alignment...
    Sequence #1: 13
    Sequence #2: 29
    Sequence #3: 25
    Sequence #4: 28
    Sequence #5: 26
    Sequence #6: 27
    Sequence #7: 33
    Sequence #8: 34
    Sequence #9: 8
```

```
    Sequence #10: 11
    Sequence #11: 6
    Sequence #12: 3
    Sequence #13: 9
    Sequence #14: 19
    Sequence #15: 24
    Sequence #16: 22
    Sequence #17: 23
    Sequence #18: 32
    Sequence #19: 30
    Sequence #20: 31
    Sequence #21: 35
    Sequence #22: 36
    Sequence #23: 4
    Sequence #24: 12
    Sequence #25: 17
    Sequence #26: 18
    Sequence #27: 14
    Sequence #28: 15
    Sequence #29: 16
    Sequence #30: 1
    Sequence #31: 2
    Sequence #32: 7
    Sequence #33: 10
    Sequence #34: 5
    Sequence #35: 20
    Sequence #36: 21
   Alignment contains 36 sequences of length 595

**********************************************************
Observed number of invariant sites: 0
Observed aminoacid frequencies:
 A: 0.106    C: 0.017    D: 0.053    E: 0.058    F: 0.038
 G: 0.081    H: 0.020    I: 0.052    K: 0.069    L: 0.101
 M: 0.021    N: 0.028    P: 0.051    Q: 0.024    R: 0.036
 S: 0.068    T: 0.057    V: 0.089    W: 0.007    Y: 0.024
**********************************************************

Model................................ : JTT
  Number of parameters............... : 69 (0 + 69 branch length
estimates)
 -lnL................................ = 16297.79     (0h0m5s)

Model................................ : JTT+F
  Number of parameters............... : 88 (19 + 69 branch length
estimates)
    aminoacid frequencies............ = observed (see above)
 -lnL................................ = 16281.35     (0h0m4s)

Model................................ : JTT+I
  Number of parameters............... : 70 (1 + 69 branch length
estimates)
    proportion of invariable sites... = 0.005
 -lnL................................ = 16286.56     (0h0m10s)

Model................................ : JTT+I+F
  Number of parameters............... : 89 (20 + 69 branch length
estimates)
    proportion of invariable sites... = 0.005
    aminoacid frequencies............ = observed (see above)
```

```
 -lnL................................ = 16269.33     (0h0m13s)

Model................................ : JTT+G
  Number of parameters............... : 70 (1 + 69 branch length
estimates)
    gamma shape (4 rate categories).. = 3.584
 -lnL................................ = 16148.58     (0h2m3s)

Model................................ : JTT+G+F
  Number of parameters............... : 89 (20 + 69 branch length
estimates)
    gamma shape (4 rate categories).. = 3.341
    aminoacid frequencies............ = observed (see above)
 -lnL................................ = 16126.67     (0h0m21s)

Model................................ : JTT+I+G
  Number of parameters............... : 71 (2 + 69 branch length
estimates)
    gamma shape (4 rate categories).. = 3.715
    proportion of invariable sites... = 0.003
 -lnL................................ = 16147.51     (0h0m53s)

Model................................ : JTT+I+G+F
  Number of parameters............... : 90 (21 + 69 branch length
estimates)
    gamma shape (4 rate categories).. = 3.462
    proportion of invariable sites... = 0.003
    aminoacid frequencies............ = observed (see above)
 -lnL................................ = 16125.68     (0h1m11s)

Model................................ : LG
  Number of parameters............... : 69 (0 + 69 branch length
estimates)
 -lnL................................ = 16277.64     (0h0m17s)

Model................................ : LG+F
  Number of parameters............... : 88 (19 + 69 branch length
estimates)
    aminoacid frequencies............ = observed (see above)
 -lnL................................ = 16228.09     (0h0m21s)

Model................................ : LG+I
  Number of parameters............... : 70 (1 + 69 branch length
estimates)
    proportion of invariable sites... = 0.004
 -lnL................................ = 16271.03     (0h0m19s)

Model................................ : LG+I+F
  Number of parameters............... : 89 (20 + 69 branch length
estimates)
    proportion of invariable sites... = 0.004
    aminoacid frequencies............ = observed (see above)
 -lnL................................ = 16221.52     (0h0m19s)

Model................................ : LG+G
  Number of parameters............... : 70 (1 + 69 branch length
estimates)
    gamma shape (4 rate categories).. = 3.207
 -lnL................................ = 16176.17     (0h1m9s)
```

```
Model................................ : LG+G+F
  Number of parameters............... : 89 (20 + 69 branch length
estimates)
    gamma shape (4 rate categories).. = 3.107
    aminoacid frequencies............ = observed (see above)
 -lnL................................ = 16120.43     (0h0m21s)

Model................................ : LG+I+G
  Number of parameters............... : 71 (2 + 69 branch length
estimates)
    gamma shape (4 rate categories).. = 3.327
    proportion of invariable sites... = 0.003
 -lnL................................ = 16175.21     (0h0m56s)

Model................................ : LG+I+G+F
  Number of parameters............... : 90 (21 + 69 branch length
estimates)
    gamma shape (4 rate categories).. = 3.2
    proportion of invariable sites... = 0.003
    aminoacid frequencies............ = observed (see above)
 -lnL................................ = 16119.73     (0h3m40s)

Model................................ : DCMut
  Number of parameters............... : 69 (0 + 69 branch length
estimates)
 -lnL................................ = 16379.06     (0h0m15s)

Model................................ : DCMut+F
  Number of parameters............... : 88 (19 + 69 branch length
estimates)
    aminoacid frequencies............ = observed (see above)
 -lnL................................ = 16365.41     (0h0m33s)

Model................................ : DCMut+I
  Number of parameters............... : 70 (1 + 69 branch length
estimates)
    proportion of invariable sites... = 0.005
 -lnL................................ = 16368.10     (0h0m14s)

Model................................ : DCMut+I+F
  Number of parameters............... : 89 (20 + 69 branch length
estimates)
    proportion of invariable sites... = 0.005
    aminoacid frequencies............ = observed (see above)
 -lnL................................ = 16354.90     (0h0m26s)

Model................................ : DCMut+G
  Number of parameters............... : 70 (1 + 69 branch length
estimates)
    gamma shape (4 rate categories).. = 3.435
 -lnL................................ = 16279.67     (0h1m51s)

Model................................ : DCMut+G+F
  Number of parameters............... : 89 (20 + 69 branch length
estimates)
    gamma shape (4 rate categories).. = 3.176
    aminoacid frequencies............ = observed (see above)
 -lnL................................ = 16258.18     (0h0m22s)

Model................................ : DCMut+I+G
```

```
  Number of parameters.............. : 71 (2 + 69 branch length
estimates)
    gamma shape (4 rate categories).. = 3.619
    proportion of invariable sites... = 0.003
 -lnL............................... = 16276.39     (0h1m0s)

Model............................... : DCMut+I+G+F
  Number of parameters.............. : 90 (21 + 69 branch length
estimates)
    gamma shape (4 rate categories).. = 3.322
    proportion of invariable sites... = 0.003
    aminoacid frequencies........... = observed (see above)
 -lnL............................... = 16255.41     (0h4m44s)

Model............................... : MtREV
  Number of parameters.............. : 69 (0 + 69 branch length
estimates)
 -lnL............................... = 17139.91     (0h0m3s)

Model............................... : MtREV+F
  Number of parameters.............. : 88 (19 + 69 branch length
estimates)
    aminoacid frequencies........... = observed (see above)
 -lnL............................... = 16427.52     (0h0m4s)

Model............................... : MtREV+I
  Number of parameters.............. : 70 (1 + 69 branch length
estimates)
    proportion of invariable sites... = 0.004
 -lnL............................... = 17136.63     (0h0m10s)

Model............................... : MtREV+I+F
  Number of parameters.............. : 89 (20 + 69 branch length
estimates)
    proportion of invariable sites... = 0.004
    aminoacid frequencies........... = observed (see above)
 -lnL............................... = 16419.31     (0h0m11s)

Model............................... : MtREV+G
  Number of parameters.............. : 70 (1 + 69 branch length
estimates)
    gamma shape (4 rate categories).. = 2.42
 -lnL............................... = 16966.08     (0h0m18s)

Model............................... : MtREV+G+F
  Number of parameters.............. : 89 (20 + 69 branch length
estimates)
    gamma shape (4 rate categories).. = 2.602
    aminoacid frequencies........... = observed (see above)
 -lnL............................... = 16287.10     (0h0m21s)

Model............................... : MtREV+I+G
  Number of parameters.............. : 71 (2 + 69 branch length
estimates)
    gamma shape (4 rate categories).. = 2.43
    proportion of invariable sites... = 0.001
 -lnL............................... = 16966.04     (0h2m30s)

Model............................... : MtREV+I+G+F
```

```
  Number of parameters.............. : 90 (21 + 69 branch length
estimates)
    gamma shape (4 rate categories).. = 2.417
    proportion of invariable sites... = 0.003
    aminoacid frequencies........... = observed (see above)
 -lnL................................ = 16293.94     (0h1m31s)

Model................................ : MtMam
  Number of parameters.............. : 69 (0 + 69 branch length
estimates)
 -lnL................................ = 17715.77     (0h0m16s)

Model................................ : MtMam+F
  Number of parameters.............. : 88 (19 + 69 branch length
estimates)
    aminoacid frequencies........... = observed (see above)
 -lnL................................ = 16927.47     (0h0m5s)

Model................................ : MtMam+I
  Number of parameters.............. : 70 (1 + 69 branch length
estimates)
    proportion of invariable sites... = 0.004
 -lnL................................ = 17709.26     (0h0m13s)

Model................................ : MtMam+I+F
  Number of parameters.............. : 89 (20 + 69 branch length
estimates)
    proportion of invariable sites... = 0.005
    aminoacid frequencies........... = observed (see above)
 -lnL................................ = 16913.42     (0h0m16s)

Model................................ : MtMam+G
  Number of parameters.............. : 70 (1 + 69 branch length
estimates)
    gamma shape (4 rate categories).. = 1.24
 -lnL................................ = 17332.79     (0h0m30s)

Model................................ : MtMam+G+F
  Number of parameters.............. : 89 (20 + 69 branch length
estimates)
    gamma shape (4 rate categories).. = 1.411
    aminoacid frequencies........... = observed (see above)
 -lnL................................ = 16606.40     (0h0m27s)

Model................................ : MtMam+I+G
  Number of parameters.............. : 71 (2 + 69 branch length
estimates)
    gamma shape (4 rate categories).. = 1.251
    proportion of invariable sites... = 0.002
 -lnL................................ = 17329.75     (0h3m17s)

Model................................ : MtMam+I+G+F
  Number of parameters.............. : 90 (21 + 69 branch length
estimates)
    gamma shape (4 rate categories).. = 1.42
    proportion of invariable sites... = 0.003
    aminoacid frequencies........... = observed (see above)
 -lnL................................ = 16603.09     (0h2m59s)

Model................................ : MtArt
```

```
  Number of parameters............... : 69 (0 + 69 branch length
estimates)
 -lnL................................ = 17721.82     (0h0m11s)

Model................................ : MtArt+F
  Number of parameters............... : 88 (19 + 69 branch length
estimates)
    aminoacid frequencies............ = observed (see above)
 -lnL................................ = 16974.71     (0h0m4s)

Model................................ : MtArt+I
  Number of parameters............... : 70 (1 + 69 branch length
estimates)
    proportion of invariable sites... = 0.005
 -lnL................................ = 17714.65     (0h0m13s)

Model................................ : MtArt+I+F
  Number of parameters............... : 89 (20 + 69 branch length
estimates)
    proportion of invariable sites... = 0.004
    aminoacid frequencies............ = observed (see above)
 -lnL................................ = 16964.75     (0h0m11s)

Model................................ : MtArt+G
  Number of parameters............... : 70 (1 + 69 branch length
estimates)
    gamma shape (4 rate categories).. = 1.336
 -lnL................................ = 17352.93     (0h0m25s)

Model................................ : MtArt+G+F
  Number of parameters............... : 89 (20 + 69 branch length
estimates)
    gamma shape (4 rate categories).. = 1.321
    aminoacid frequencies............ = observed (see above)
 -lnL................................ = 16676.22     (0h0m24s)

Model................................ : MtArt+I+G
  Number of parameters............... : 71 (2 + 69 branch length
estimates)
    gamma shape (4 rate categories).. = 1.344
    proportion of invariable sites... = 0.002
 -lnL................................ = 17351.59     (0h1m1s)

Model................................ : MtArt+I+G+F
  Number of parameters............... : 90 (21 + 69 branch length
estimates)
    gamma shape (4 rate categories).. = 1.328
    proportion of invariable sites... = 0.003
    aminoacid frequencies............ = observed (see above)
 -lnL................................ = 16674.88     (0h2m20s)

Model................................ : Dayhoff
  Number of parameters............... : 69 (0 + 69 branch length
estimates)
 -lnL................................ = 16380.24     (0h0m16s)

Model................................ : Dayhoff+F
  Number of parameters............... : 88 (19 + 69 branch length
estimates)
    aminoacid frequencies............ = observed (see above)
```

```
 -lnL................................ = 16366.73     (0h0m34s)

Model................................ : Dayhoff+I
  Number of parameters............... : 70 (1 + 69 branch length
estimates)
    proportion of invariable sites... = 0.005
 -lnL................................ = 16369.22     (0h0m14s)

Model................................ : Dayhoff+I+F
  Number of parameters............... : 89 (20 + 69 branch length
estimates)
    proportion of invariable sites... = 0.005
    aminoacid frequencies............ = observed (see above)
 -lnL................................ = 16356.17     (0h0m29s)

Model................................ : Dayhoff+G
  Number of parameters............... : 70 (1 + 69 branch length
estimates)
    gamma shape (4 rate categories).. = 3.418
 -lnL................................ = 16280.17     (0h1m53s)

Model................................ : Dayhoff+G+F
  Number of parameters............... : 89 (20 + 69 branch length
estimates)
    gamma shape (4 rate categories).. = 3.16
    aminoacid frequencies............ = observed (see above)
 -lnL................................ = 16258.78     (0h0m22s)

Model................................ : Dayhoff+I+G
  Number of parameters............... : 71 (2 + 69 branch length
estimates)
    gamma shape (4 rate categories).. = 3.599
    proportion of invariable sites... = 0.003
 -lnL................................ = 16276.88     (0h1m1s)

Model................................ : Dayhoff+I+G+F
  Number of parameters............... : 90 (21 + 69 branch length
estimates)
    gamma shape (4 rate categories).. = 3.305
    proportion of invariable sites... = 0.003
    aminoacid frequencies............ = observed (see above)
 -lnL................................ = 16256.00     (0h4m34s)

Model................................ : WAG
  Number of parameters............... : 69 (0 + 69 branch length
estimates)
 -lnL................................ = 16236.35     (0h0m3s)

Model................................ : WAG+F
  Number of parameters............... : 88 (19 + 69 branch length
estimates)
    aminoacid frequencies............ = observed (see above)
 -lnL................................ = 16207.18     (0h0m4s)

Model................................ : WAG+I
  Number of parameters............... : 70 (1 + 69 branch length
estimates)
    proportion of invariable sites... = 0.005
 -lnL................................ = 16222.18     (0h0m10s)
```

```
Model................................ : WAG+I+F
  Number of parameters............... : 89 (20 + 69 branch length
estimates)
    proportion of invariable sites... = 0.005
    aminoacid frequencies............ = observed (see above)
 -lnL................................ = 16192.85     (0h0m11s)

Model................................ : WAG+G
  Number of parameters............... : 70 (1 + 69 branch length
estimates)
    gamma shape (4 rate categories).. = 4.64
 -lnL................................ = 16109.54     (0h1m48s)

Model................................ : WAG+G+F
  Number of parameters............... : 89 (20 + 69 branch length
estimates)
    gamma shape (4 rate categories).. = 4.49
    aminoacid frequencies............ = observed (see above)
 -lnL................................ = 16078.94     (0h0m42s)

Model................................ : WAG+I+G
  Number of parameters............... : 71 (2 + 69 branch length
estimates)
    gamma shape (4 rate categories).. = 4.951
    proportion of invariable sites... = 0.003
 -lnL................................ = 16106.09     (0h1m37s)

Model................................ : WAG+I+G+F
  Number of parameters............... : 90 (21 + 69 branch length
estimates)
    gamma shape (4 rate categories).. = 4.781
    proportion of invariable sites... = 0.003
    aminoacid frequencies............ = observed (see above)
 -lnL................................ = 16075.91     (0h2m12s)

Model................................ : RtREV
  Number of parameters............... : 69 (0 + 69 branch length
estimates)
 -lnL................................ = 16445.35     (0h0m14s)

Model................................ : RtREV+F
  Number of parameters............... : 88 (19 + 69 branch length
estimates)
    aminoacid frequencies............ = observed (see above)
 -lnL................................ = 16278.29     (0h0m27s)

Model................................ : RtREV+I
  Number of parameters............... : 70 (1 + 69 branch length
estimates)
    proportion of invariable sites... = 0.004
 -lnL................................ = 16437.57     (0h0m16s)

Model................................ : RtREV+I+F
  Number of parameters............... : 89 (20 + 69 branch length
estimates)
    proportion of invariable sites... = 0.004
    aminoacid frequencies............ = observed (see above)
 -lnL................................ = 16270.06     (0h0m24s)

Model................................ : RtREV+G
```

```
  Number of parameters.............. : 70 (1 + 69 branch length
estimates)
    gamma shape (4 rate categories).. = 3.48
 -lnL................................ = 16350.87     (0h1m21s)

Model................................ : RtREV+G+F
  Number of parameters.............. : 89 (20 + 69 branch length
estimates)
    gamma shape (4 rate categories).. = 3.199
    aminoacid frequencies............ = observed (see above)
 -lnL................................ = 16171.27     (0h0m21s)

Model................................ : RtREV+I+G
  Number of parameters.............. : 71 (2 + 69 branch length
estimates)
    gamma shape (4 rate categories).. = 3.636
    proportion of invariable sites... = 0.003
 -lnL................................ = 16349.36     (0h0m49s)

Model................................ : RtREV+I+G+F
  Number of parameters.............. : 90 (21 + 69 branch length
estimates)
    gamma shape (4 rate categories).. = 3.324
    proportion of invariable sites... = 0.003
    aminoacid frequencies............ = observed (see above)
 -lnL................................ = 16169.84     (0h4m52s)

Model................................ : CpREV
  Number of parameters.............. : 69 (0 + 69 branch length
estimates)
 -lnL................................ = 16362.37     (0h0m20s)

Model................................ : CpREV+F
  Number of parameters.............. : 88 (19 + 69 branch length
estimates)
    aminoacid frequencies............ = observed (see above)
 -lnL................................ = 16297.14     (0h0m3s)

Model................................ : CpREV+I
  Number of parameters.............. : 70 (1 + 69 branch length
estimates)
    proportion of invariable sites... = 0.004
 -lnL................................ = 16351.96     (0h0m14s)

Model................................ : CpREV+I+F
  Number of parameters.............. : 89 (20 + 69 branch length
estimates)
    proportion of invariable sites... = 0.005
    aminoacid frequencies............ = observed (see above)
 -lnL................................ = 16282.26     (0h0m32s)

Model................................ : CpREV+G
  Number of parameters.............. : 70 (1 + 69 branch length
estimates)
    gamma shape (4 rate categories).. = 3.751
 -lnL................................ = 16281.79     (0h0m21s)

Model................................ : CpREV+G+F
  Number of parameters.............. : 89 (20 + 69 branch length
estimates)
```

```
    gamma shape (4 rate categories).. = 3.73
    aminoacid frequencies........... = observed (see above)
 -lnL.............................. = 16158.79     (0h0m21s)

Model.............................. : CpREV+I+G
  Number of parameters............. : 71 (2 + 69 branch length
estimates)
    gamma shape (4 rate categories).. = 3.962
    proportion of invariable sites... = 0.003
 -lnL.............................. = 16278.85     (0h3m39s)

Model.............................. : CpREV+I+G+F
  Number of parameters............. : 90 (21 + 69 branch length
estimates)
    gamma shape (4 rate categories).. = 3.952
    proportion of invariable sites... = 0.003
    aminoacid frequencies........... = observed (see above)
 -lnL.............................. = 16155.18     (0h2m23s)

Model.............................. : Blosum62
  Number of parameters............. : 69 (0 + 69 branch length
estimates)
 -lnL.............................. = 16378.18     (0h0m3s)

Model.............................. : Blosum62+F
  Number of parameters............. : 88 (19 + 69 branch length
estimates)
    aminoacid frequencies........... = observed (see above)
 -lnL.............................. = 16315.03     (0h0m4s)

Model.............................. : Blosum62+I
  Number of parameters............. : 70 (1 + 69 branch length
estimates)
    proportion of invariable sites... = 0.005
 -lnL.............................. = 16365.45     (0h0m10s)

Model.............................. : Blosum62+I+F
  Number of parameters............. : 89 (20 + 69 branch length
estimates)
    proportion of invariable sites... = 0.005
    aminoacid frequencies........... = observed (see above)
 -lnL.............................. = 16302.12     (0h0m11s)

Model.............................. : Blosum62+G
  Number of parameters............. : 70 (1 + 69 branch length
estimates)
    gamma shape (4 rate categories).. = 4.825
 -lnL.............................. = 16247.01     (0h0m21s)

Model.............................. : Blosum62+G+F
  Number of parameters............. : 89 (20 + 69 branch length
estimates)
    gamma shape (4 rate categories).. = 4.685
    aminoacid frequencies........... = observed (see above)
 -lnL.............................. = 16186.33     (0h0m21s)

Model.............................. : Blosum62+I+G
  Number of parameters............. : 71 (2 + 69 branch length
estimates)
    gamma shape (4 rate categories).. = 5.119
```

```
    proportion of invariable sites... = 0.003
 -lnL................................ = 16244.81     (0h1m58s)

Model................................ : Blosum62+I+G+F
  Number of parameters............... : 90 (21 + 69 branch length
estimates)
    gamma shape (4 rate categories).. = 4.939
    proportion of invariable sites... = 0.003
    aminoacid frequencies............ = observed (see above)
 -lnL................................ = 16184.38     (0h4m13s)

Model................................ : VT
  Number of parameters............... : 69 (0 + 69 branch length
estimates)
 -lnL................................ = 16465.67     (0h0m4s)

Model................................ : VT+F
  Number of parameters............... : 88 (19 + 69 branch length
estimates)
    aminoacid frequencies............ = observed (see above)
 -lnL................................ = 16505.27     (0h0m5s)

Model................................ : VT+I
  Number of parameters............... : 70 (1 + 69 branch length
estimates)
    proportion of invariable sites... = 0.003
 -lnL................................ = 16459.84     (0h0m13s)

Model................................ : VT+I+F
  Number of parameters............... : 89 (20 + 69 branch length
estimates)
    proportion of invariable sites... = 0.003
    aminoacid frequencies............ = observed (see above)
 -lnL................................ = 16498.97     (0h1m13s)

Model................................ : VT+G
  Number of parameters............... : 70 (1 + 69 branch length
estimates)
    gamma shape (4 rate categories).. = 2.84
 -lnL................................ = 16342.28     (0h0m21s)

Model................................ : VT+G+F
  Number of parameters............... : 89 (20 + 69 branch length
estimates)
    gamma shape (4 rate categories).. = 2.707
    aminoacid frequencies............ = observed (see above)
 -lnL................................ = 16373.68     (0h0m21s)

Model................................ : VT+I+G
  Number of parameters............... : 71 (2 + 69 branch length
estimates)
    gamma shape (4 rate categories).. = 2.9
    proportion of invariable sites... = 0.002
 -lnL................................ = 16341.82     (0h3m13s)

Model................................ : VT+I+G+F
  Number of parameters............... : 90 (21 + 69 branch length
estimates)
    gamma shape (4 rate categories).. = 2.766
    proportion of invariable sites... = 0.002
```

```
    aminoacid frequencies........... = observed (see above)
 -lnL............................... = 16373.18     (0h2m3s)

Model............................... : HIVb
  Number of parameters.............. : 69 (0 + 69 branch length
estimates)
 -lnL............................... = 16716.36     (0h0m32s)

Model............................... : HIVb+F
  Number of parameters.............. : 88 (19 + 69 branch length
estimates)
    aminoacid frequencies........... = observed (see above)
 -lnL............................... = 16579.21     (0h0m3s)

Model............................... : HIVb+I
  Number of parameters.............. : 70 (1 + 69 branch length
estimates)
    proportion of invariable sites... = 0.005
 -lnL............................... = 16710.80     (0h0m27s)

Model............................... : HIVb+I+F
  Number of parameters.............. : 89 (20 + 69 branch length
estimates)
    proportion of invariable sites... = 0.005
    aminoacid frequencies........... = observed (see above)
 -lnL............................... = 16566.59     (0h0m14s)

Model............................... : HIVb+G
  Number of parameters.............. : 70 (1 + 69 branch length
estimates)
    gamma shape (4 rate categories).. = 2.799
 -lnL............................... = 16581.90     (0h0m34s)

Model............................... : HIVb+G+F
  Number of parameters.............. : 89 (20 + 69 branch length
estimates)
    gamma shape (4 rate categories).. = 2.485
    aminoacid frequencies........... = observed (see above)
 -lnL............................... = 16377.12     (0h0m21s)

Model............................... : HIVb+I+G
  Number of parameters.............. : 71 (2 + 69 branch length
estimates)
    gamma shape (4 rate categories).. = 2.855
    proportion of invariable sites... = 0.002
 -lnL............................... = 16581.66     (0h1m27s)

Model............................... : HIVb+I+G+F
  Number of parameters.............. : 90 (21 + 69 branch length
estimates)
    gamma shape (4 rate categories).. = 2.56
    proportion of invariable sites... = 0.003
    aminoacid frequencies........... = observed (see above)
 -lnL............................... = 16376.23     (0h1m3s)

Model............................... : HIVw
  Number of parameters.............. : 69 (0 + 69 branch length
estimates)
 -lnL............................... = 17334.21     (0h0m3s)
```

```
Model................................ : HIVw+F
  Number of parameters............... : 88 (19 + 69 branch length
estimates)
    aminoacid frequencies............ = observed (see above)
 -lnL................................ = 16879.40     (0h0m4s)

Model................................ : HIVw+I
  Number of parameters............... : 70 (1 + 69 branch length
estimates)
    proportion of invariable sites... = 0.006
 -lnL................................ = 17322.53     (0h0m11s)

Model................................ : HIVw+I+F
  Number of parameters............... : 89 (20 + 69 branch length
estimates)
    proportion of invariable sites... = 0.007
    aminoacid frequencies............ = observed (see above)
 -lnL................................ = 16864.58     (0h0m10s)

Model................................ : HIVw+G
  Number of parameters............... : 70 (1 + 69 branch length
estimates)
    gamma shape (4 rate categories).. = 2.411
 -lnL................................ = 17174.49     (0h0m19s)

Model................................ : HIVw+G+F
  Number of parameters............... : 89 (20 + 69 branch length
estimates)
    gamma shape (4 rate categories).. = 2.167
    aminoacid frequencies............ = observed (see above)
 -lnL................................ = 16708.13     (0h0m17s)

Model................................ : HIVw+I+G
  Number of parameters............... : 71 (2 + 69 branch length
estimates)
    gamma shape (4 rate categories).. = 2.513
    proportion of invariable sites... = 0.004
 -lnL................................ = 17172.00     (0h1m14s)

Model................................ : HIVw+I+G+F
  Number of parameters............... : 90 (21 + 69 branch length
estimates)
    gamma shape (4 rate categories).. = 2.272
    proportion of invariable sites... = 0.004
    aminoacid frequencies............ = observed (see above)
 -lnL................................ = 16704.02     (0h4m55s)

************************************************************
Date   :  Wed Oct 19 18:30:53 CEST 2016
Runtime:  1h:39:25


************************************************************
Best model according to -lnL: WAG+I+G+F
************************************************************
Model         deltaAIC     AIC          -lnL*        AICw
------------------------------------------------------------
WAG+I+G+F     0.00         32331.82     -16075.91    0.88
WAG+G+F       4.07         32335.88     -16078.94    0.12
WAG+I+G       22.37        32354.19     -16106.09    0.00
```

WAG+G	27.27	32359.09	-16109.54	0.00
LG+I+G+F	87.64	32419.46	-16119.73	0.00
LG+G+F	87.03	32418.85	-16120.43	0.00
JTT+I+G+F	99.54	32431.36	-16125.68	0.00
JTT+G+F	99.51	32431.33	-16126.67	0.00
JTT+I+G	105.21	32437.02	-16147.51	0.00
JTT+G	105.35	32437.17	-16148.58	0.00
CpREV+I+G+F	158.54	32490.35	-16155.18	0.00
CpREV+G+F	163.76	32495.58	-16158.79	0.00
RtREV+I+G+F	187.85	32519.67	-16169.84	0.00
RtREV+G+F	188.71	32520.53	-16171.27	0.00
LG+I+G	160.60	32492.42	-16175.21	0.00
LG+G	160.53	32492.35	-16176.17	0.00
Blosum62+I+G+F216.94		32548.76	-16184.38	0.00
Blosum62+G+F	218.85	32550.67	-16186.33	0.00
WAG+I+F	231.87	32563.69	-16192.85	0.00
WAG+F	258.54	32590.36	-16207.18	0.00
LG+I+F	289.21	32621.03	-16221.52	0.00
WAG+I	252.54	32584.36	-16222.18	0.00
LG+F	300.36	32632.18	-16228.09	0.00
WAG	278.89	32610.71	-16236.35	0.00
Blosum62+I+G	299.80	32631.62	-16244.81	0.00
Blosum62+G	302.19	32634.01	-16247.01	0.00
DCMut+I+G+F	359.01	32690.83	-16255.41	0.00
Dayhoff+I+G+F360.18		32692.00	-16256.00	0.00
DCMut+G+F	362.54	32694.36	-16258.18	0.00
Dayhoff+G+F	363.74	32695.56	-16258.78	0.00
JTT+I+F	384.84	32716.66	-16269.33	0.00
RtREV+I+F	386.29	32718.11	-16270.06	0.00
LG+I	350.24	32682.06	-16271.03	0.00
DCMut+I+G	362.96	32694.78	-16276.39	0.00
Dayhoff+I+G	363.93	32695.75	-16276.88	0.00
LG	361.47	32693.29	-16277.64	0.00
RtREV+F	400.76	32732.58	-16278.29	0.00
CpREV+I+G	367.88	32699.70	-16278.85	0.00
DCMut+G	367.52	32699.34	-16279.67	0.00
Dayhoff+G	368.52	32700.33	-16280.17	0.00
JTT+F	406.87	32738.69	-16281.35	0.00
CpREV+G	371.77	32703.59	-16281.79	0.00
CpREV+I+F	410.70	32742.52	-16282.26	0.00
JTT+I	381.29	32713.11	-16286.56	0.00
MtREV+G+F	420.37	32752.19	-16287.10	0.00
MtREV+I+G+F	436.05	32767.87	-16293.94	0.00
CpREV+F	438.46	32770.28	-16297.14	0.00
JTT	401.77	32733.59	-16297.79	0.00
Blosum62+I+F	450.43	32782.25	-16302.12	0.00
Blosum62+F	474.25	32806.07	-16315.03	0.00
VT+I+G	493.82	32825.63	-16341.82	0.00
VT+G	492.74	32824.56	-16342.28	0.00
RtREV+I+G	508.89	32840.71	-16349.36	0.00
RtREV+G	509.92	32841.74	-16350.87	0.00
CpREV+I	512.10	32843.92	-16351.96	0.00
DCMut+I+F	555.98	32887.80	-16354.90	0.00
Dayhoff+I+F	558.53	32890.35	-16356.17	0.00
CpREV	530.92	32862.74	-16362.37	0.00
DCMut+F	575.00	32906.82	-16365.41	0.00
Blosum62+I	539.08	32870.90	-16365.45	0.00
Dayhoff+F	577.63	32909.45	-16366.73	0.00
DCMut+I	544.37	32876.19	-16368.10	0.00
Dayhoff+I	546.62	32878.44	-16369.22	0.00

VT+I+G+F	594.53	32926.35	-16373.18	0.00
VT+G+F	593.53	32925.35	-16373.68	0.00
HIVb+I+G+F	600.63	32932.45	-16376.23	0.00
HIVb+G+F	600.43	32932.25	-16377.12	0.00
Blosum62	562.54	32894.36	-16378.18	0.00
DCMut	564.31	32896.13	-16379.06	0.00
Dayhoff	566.65	32898.47	-16380.24	0.00
MtREV+I+F	684.80	33016.62	-16419.31	0.00
MtREV+F	699.21	33031.03	-16427.52	0.00
RtREV+I	683.32	33015.14	-16437.57	0.00
RtREV	696.88	33028.70	-16445.35	0.00
VT+I	727.87	33059.69	-16459.84	0.00
VT	737.51	33069.33	-16465.67	0.00
VT+I+F	844.13	33175.95	-16498.97	0.00
VT+F	854.73	33186.54	-16505.27	0.00
HIVb+I+F	979.36	33311.18	-16566.59	0.00
HIVb+F	1002.60	33334.42	-16579.21	0.00
HIVb+I+G	973.49	33305.31	-16581.66	0.00
HIVb+G	971.98	33303.80	-16581.90	0.00
MtMam+I+G+F	1054.36	33386.17	-16603.09	0.00
MtMam+G+F	1058.98	33390.80	-16606.40	0.00
MtArt+I+G+F	1197.94	33529.76	-16674.88	0.00
MtArt+G+F	1198.63	33530.45	-16676.22	0.00
HIVw+I+G+F	1256.22	33588.04	-16704.02	0.00
HIVw+G+F	1262.43	33594.25	-16708.13	0.00
HIVb+I	1229.78	33561.60	-16710.80	0.00
HIVb	1238.90	33570.72	-16716.36	0.00
HIVw+I+F	1575.35	33907.17	-16864.58	0.00
HIVw+F	1602.98	33934.80	-16879.40	0.00
MtMam+I+F	1673.02	34004.84	-16913.42	0.00
MtMam+F	1699.13	34030.95	-16927.47	0.00
MtArt+I+F	1775.67	34107.49	-16964.75	0.00
MtREV+I+G	1742.27	34074.09	-16966.04	0.00
MtREV+G	1740.33	34072.15	-16966.08	0.00
MtArt+F	1793.60	34125.42	-16974.71	0.00
MtREV+I	2081.43	34413.25	-17136.63	0.00
MtREV	2086.01	34417.83	-17139.91	0.00
HIVw+I+G	2154.17	34485.99	-17172.00	0.00
HIVw+G	2157.16	34488.98	-17174.49	0.00
HIVw+I	2453.24	34785.06	-17322.53	0.00
MtMam+I+G	2469.69	34801.51	-17329.75	0.00
MtMam+G	2473.76	34805.58	-17332.79	0.00
HIVw	2474.59	34806.41	-17334.21	0.00
MtArt+I+G	2513.37	34845.19	-17351.59	0.00
MtArt+G	2514.04	34845.86	-17352.93	0.00
MtMam+I	3226.69	35558.51	-17709.26	0.00
MtArt+I	3237.47	35569.29	-17714.65	0.00
MtMam	3237.72	35569.54	-17715.77	0.00
MtArt	3249.81	35581.63	-17721.82	0.00

----
*: models sorted according to this column

****
Tree according to best model (WAG+I+G+F)
((((((((2:1.3376343,1:0.0000034):2.4908945,13:0.0000169):1.8480518,(((
((((21:0.0373925,20:0.0179435):0.2034483,(10:0.1592520,7:0.0715029):0.241
1376)
:0.0696539,5:0.2297576):0.1144497,(((((16:0.0000007,15:0.0092216):0.07470
62,

14:0.0000000):0.0760066,(18:0.0000000,17:0.0000001):0.0408802):0.2094018,
12:0.3716217):0.0955446,4:0.3412932):0.3830861):0.4426901,((36:0.1591337,
35:0.3648360):0.2157303,(31:0.4673746,30:0.4165845):0.0657238):0.2350085)
:0.2422752,32:0.9136842):0.3226214,(((((23:0.0130404,22:0.0000002):0.0191
199,
19:0.1113775):0.0340081,24:0.2356387):0.1084668,9:0.1566060):0.1002199,
(6:0.4089991,(11:0.1858990,8:0.1201067):0.4137185):0.4083163):0.9618768)
:0.6631195):0.1133725,3:6.0061763):0.3501338,(34:0.2695394,33:0.3173625)
:0.2124186):0.3700485,29:0.2619367):0.2580275,25:0.0798008):0.3302370,27:
0.0042595):0.0000002,26:0.0000004,28:0.0042595);
****

Table: Weights(Ranking) of the candidate models under the different frameworks

model	AIC	AICc-1	AICc-2	AICc-3	BIC-1	BIC-2	BIC-3
WAG+I+G+F	0.88(1)	0.83(1)	0.86(1)	0.88(1)	0.00(4)	0.00(4)	0.00(6)
WAG+G+F	0.12(2)	0.16(2)	0.14(2)	0.12(2)	0.00(3)	0.00(3)	0.00(5)
WAG+I+G	0.00(3)	0.01(3)	0.00(3)	0.00(3)	0.56(1)	0.49(2)	0.25(2)
WAG+G	0.00(4)	0.00(4)	0.00(4)	0.00(4)	0.44(2)	0.51(1)	0.75(1)
LG+G+F	0.00(5)	0.00(5)	0.00(5)	0.00(5)	0.00(9)	0.00(9)	0.00(9)
LG+I+G+F	0.00(6)	0.00(6)	0.00(6)	0.00(6)	0.00(10)	0.00(10)	0.00(10)
JTT+G+F	0.00(7)	0.00(9)	0.00(9)	0.00(7)	0.00(11)	0.00(11)	0.00(11)
JTT+I+G+F	0.00(8)	0.00(10)	0.00(10)	0.00(8)	0.00(12)	0.00(12)	0.00(12)
JTT+I+G	0.00(9)	0.00(8)	0.00(8)	0.00(9)	0.00(6)	0.00(6)	0.00(4)
JTT+G	0.00(10)	0.00(7)	0.00(7)	0.00(10)	0.00(5)	0.00(5)	0.00(3)
CpREV+I+G+F	0.00(11)	0.00(13)	0.00(13)	0.00(11)	0.00(13)	0.00(14)	0.00(16)
LG+G	0.00(12)	0.00(11)	0.00(11)	0.00(12)	0.00(7)	0.00(7)	0.00(7)
LG+I+G	0.00(13)	0.00(12)	0.00(12)	0.00(13)	0.00(8)	0.00(8)	0.00(8)
CpREV+G+F	0.00(14)	0.00(14)	0.00(14)	0.00(14)	0.00(14)	0.00(15)	0.00(15)
RtREV+I+G+F	0.00(15)	0.00(15)	0.00(15)	0.00(15)	0.00(18)	0.00(18)	0.00(20)
RtREV+G+F	0.00(16)	0.00(16)	0.00(16)	0.00(16)	0.00(16)	0.00(17)	0.00(19)
Blosum62+I+G+F	0.00(17)	0.00(17)	0.00(17)	0.00(17)	0.00(22)	0.00(22)	0.00(24)
Blosum62+G+F	0.00(18)	0.00(18)	0.00(18)	0.00(18)	0.00(20)	0.00(21)	0.00(22)
WAG+I+F	0.00(19)	0.00(19)	0.00(19)	0.00(19)	0.00(23)	0.00(23)	0.00(25)
WAG+I	0.00(20)	0.00(20)	0.00(20)	0.00(20)	0.00(15)	0.00(13)	0.00(13)

WAG+F	0.00(21)	0.00(21)	0.00(21)	0.00(21)	0.00(24)	0.00(24)	0.00(33)
WAG	0.00(22)	0.00(22)	0.00(22)	0.00(22)	0.00(17)	0.00(16)	0.00(14)
LG+I+F	0.00(23)	0.00(24)	0.00(23)	0.00(23)	0.00(33)	0.00(34)	0.00(35)
Blosum62+I+G	0.00(24)	0.00(23)	0.00(24)	0.00(24)	0.00(21)	0.00(20)	0.00(18)
LG+F	0.00(25)	0.00(26)	0.00(26)	0.00(25)	0.00(34)	0.00(35)	0.00(36)
Blosum62+G	0.00(26)	0.00(25)	0.00(25)	0.00(26)	0.00(19)	0.00(19)	0.00(17)
LG+I	0.00(27)	0.00(27)	0.00(27)	0.00(27)	0.00(25)	0.00(25)	0.00(21)
DCMut+I+G+F	0.00(28)	0.00(35)	0.00(31)	0.00(28)	0.00(38)	0.00(39)	0.00(40)
Dayhoff+I+G+F	0.00(29)	0.00(36)	0.00(32)	0.00(29)	0.00(40)	0.00(40)	0.00(42)
LG	0.00(30)	0.00(28)	0.00(28)	0.00(30)	0.00(26)	0.00(26)	0.00(23)
DCMut+G+F	0.00(31)	0.00(37)	0.00(36)	0.00(32)	0.00(37)	0.00(37)	0.00(38)
DCMut+I+G	0.00(32)	0.00(29)	0.00(29)	0.00(31)	0.00(27)	0.00(28)	0.00(28)
Dayhoff+G+F	0.00(33)	0.00(38)	0.00(37)	0.00(34)	0.00(39)	0.00(38)	0.00(39)
Dayhoff+I+G	0.00(34)	0.00(30)	0.00(30)	0.00(33)	0.00(29)	0.00(30)	0.00(29)
DCMut+G	0.00(35)	0.00(31)	0.00(33)	0.00(35)	0.00(28)	0.00(27)	0.00(26)
CpREV+I+G	0.00(36)	0.00(32)	0.00(34)	0.00(36)	0.00(32)	0.00(32)	0.00(31)
Dayhoff+G	0.00(37)	0.00(33)	0.00(35)	0.00(37)	0.00(30)	0.00(29)	0.00(27)
CpREV+G	0.00(38)	0.00(34)	0.00(38)	0.00(38)	0.00(31)	0.00(31)	0.00(30)
JTT+I	0.00(39)	0.00(39)	0.00(39)	0.00(39)	0.00(35)	0.00(33)	0.00(32)
JTT+I+F	0.00(40)	0.00(40)	0.00(40)	0.00(40)	0.00(41)	0.00(41)	0.00(46)
RtREV+I+F	0.00(41)	0.00(41)	0.00(41)	0.00(41)	0.00(42)	0.00(42)	0.00(47)
RtREV+F	0.00(42)	0.00(43)	0.00(43)	0.00(42)	0.00(43)	0.00(43)	0.00(49)
JTT	0.00(43)	0.00(42)	0.00(42)	0.00(43)	0.00(36)	0.00(36)	0.00(34)
JTT+F	0.00(44)	0.00(44)	0.00(44)	0.00(44)	0.00(44)	0.00(45)	0.00(50)
CpREV+I+F	0.00(45)	0.00(45)	0.00(45)	0.00(45)	0.00(46)	0.00(47)	0.00(53)
MtREV+G+F	0.00(46)	0.00(46)	0.00(46)	0.00(46)	0.00(48)	0.00(51)	0.00(55)
MtREV+I+G+F	0.00(47)	0.00(47)	0.00(47)	0.00(47)	0.00(53)	0.00(54)	0.00(60)
CpREV+F	0.00(48)	0.00(48)	0.00(48)	0.00(48)	0.00(52)	0.00(53)	0.00(59)
Blosum62+I+F	0.00(49)	0.00(49)	0.00(49)	0.00(49)	0.00(55)	0.00(57)	0.00(61)
Blosum62+F	0.00(50)	0.00(50)	0.00(50)	0.00(50)	0.00(59)	0.00(62)	0.00(62)

VT+G	0.00(51)	0.00(51)	0.00(51)	0.00(51)	0.00(45)	0.00(44)	0.00(37)
VT+I+G	0.00(52)	0.00(52)	0.00(52)	0.00(52)	0.00(47)	0.00(46)	0.00(41)
RtREV+I+G	0.00(53)	0.00(53)	0.00(53)	0.00(53)	0.00(51)	0.00(50)	0.00(45)
RtREV+G	0.00(54)	0.00(54)	0.00(54)	0.00(54)	0.00(49)	0.00(48)	0.00(43)
CpREV+I	0.00(55)	0.00(55)	0.00(55)	0.00(55)	0.00(50)	0.00(49)	0.00(44)
CpREV	0.00(56)	0.00(56)	0.00(56)	0.00(56)	0.00(54)	0.00(52)	0.00(48)
Blosum62+I	0.00(57)	0.00(57)	0.00(57)	0.00(57)	0.00(56)	0.00(55)	0.00(51)
DCMut+I	0.00(58)	0.00(58)	0.00(58)	0.00(58)	0.00(57)	0.00(56)	0.00(52)
Dayhoff+I	0.00(59)	0.00(59)	0.00(59)	0.00(59)	0.00(58)	0.00(58)	0.00(54)
DCMut+I+F	0.00(60)	0.00(63)	0.00(61)	0.00(60)	0.00(63)	0.00(63)	0.00(65)
Dayhoff+I+F	0.00(61)	0.00(64)	0.00(63)	0.00(61)	0.00(64)	0.00(64)	0.00(66)
Blosum62	0.00(62)	0.00(60)	0.00(60)	0.00(62)	0.00(60)	0.00(59)	0.00(56)
DCMut	0.00(63)	0.00(61)	0.00(62)	0.00(63)	0.00(61)	0.00(60)	0.00(57)
Dayhoff	0.00(64)	0.00(62)	0.00(64)	0.00(64)	0.00(62)	0.00(61)	0.00(58)
DCMut+F	0.00(65)	0.00(65)	0.00(65)	0.00(65)	0.00(65)	0.00(65)	0.00(67)
Dayhoff+F	0.00(66)	0.00(66)	0.00(66)	0.00(66)	0.00(66)	0.00(66)	0.00(68)
VT+G+F	0.00(67)	0.00(67)	0.00(67)	0.00(67)	0.00(67)	0.00(68)	0.00(70)
VT+I+G+F	0.00(68)	0.00(68)	0.00(68)	0.00(68)	0.00(68)	0.00(70)	0.00(73)
HIVb+G+F	0.00(69)	0.00(69)	0.00(69)	0.00(69)	0.00(70)	0.00(71)	0.00(72)
HIVb+I+G+F	0.00(70)	0.00(70)	0.00(70)	0.00(70)	0.00(71)	0.00(72)	0.00(74)
RtREV+I	0.00(71)	0.00(71)	0.00(71)	0.00(71)	0.00(69)	0.00(67)	0.00(63)
MtREV+I+F	0.00(72)	0.00(73)	0.00(72)	0.00(72)	0.00(75)	0.00(75)	0.00(75)
RtREV	0.00(73)	0.00(72)	0.00(73)	0.00(73)	0.00(72)	0.00(69)	0.00(64)
MtREV+F	0.00(74)	0.00(74)	0.00(74)	0.00(74)	0.00(76)	0.00(76)	0.00(76)
VT+I	0.00(75)	0.00(75)	0.00(75)	0.00(75)	0.00(73)	0.00(73)	0.00(69)
VT	0.00(76)	0.00(76)	0.00(76)	0.00(76)	0.00(74)	0.00(74)	0.00(71)
VT+I+F	0.00(77)	0.00(77)	0.00(77)	0.00(77)	0.00(77)	0.00(77)	0.00(78)
VT+F	0.00(78)	0.00(78)	0.00(78)	0.00(78)	0.00(78)	0.00(78)	0.00(80)
HIVb+G	0.00(79)	0.00(79)	0.00(79)	0.00(79)	0.00(79)	0.00(79)	0.00(77)
HIVb+I+G	0.00(80)	0.00(80)	0.00(80)	0.00(80)	0.00(80)	0.00(80)	0.00(79)

HIVb+I+F	0.00(81)	0.00(81)	0.00(81)	0.00(81)	0.00(81)	0.00(81)	0.00(81)
HIVb+F	0.00(82)	0.00(82)	0.00(82)	0.00(82)	0.00(82)	0.00(82)	0.00(82)
MtMam+I+G+F	0.00(83)	0.00(83)	0.00(83)	0.00(83)	0.00(83)	0.00(84)	0.00(84)
MtMam+G+F	0.00(84)	0.00(84)	0.00(84)	0.00(84)	0.00(84)	0.00(83)	0.00(83)
MtArt+I+G+F	0.00(85)	0.00(86)	0.00(85)	0.00(85)	0.00(88)	0.00(88)	0.00(88)
MtArt+G+F	0.00(86)	0.00(85)	0.00(86)	0.00(86)	0.00(87)	0.00(87)	0.00(87)
HIVb+I	0.00(87)	0.00(87)	0.00(87)	0.00(87)	0.00(85)	0.00(85)	0.00(85)
HIVb	0.00(88)	0.00(88)	0.00(88)	0.00(88)	0.00(86)	0.00(86)	0.00(86)
HIVw+I+G+F	0.00(89)	0.00(89)	0.00(89)	0.00(89)	0.00(89)	0.00(89)	0.00(90)
HIVw+G+F	0.00(90)	0.00(90)	0.00(90)	0.00(90)	0.00(90)	0.00(90)	0.00(89)
HIVw+I+F	0.00(91)	0.00(91)	0.00(91)	0.00(91)	0.00(91)	0.00(91)	0.00(91)
HIVw+F	0.00(92)	0.00(92)	0.00(92)	0.00(92)	0.00(92)	0.00(92)	0.00(92)
MtMam+I+F	0.00(93)	0.00(93)	0.00(93)	0.00(93)	0.00(95)	0.00(95)	0.00(95)
MtMam+F	0.00(94)	0.00(94)	0.00(94)	0.00(94)	0.00(96)	0.00(96)	0.00(96)
MtREV+G	0.00(95)	0.00(95)	0.00(95)	0.00(95)	0.00(93)	0.00(93)	0.00(93)
MtREV+I+G	0.00(96)	0.00(96)	0.00(96)	0.00(96)	0.00(94)	0.00(94)	0.00(94)
MtArt+I+F	0.00(97)	0.00(97)	0.00(97)	0.00(97)	0.00(97)	0.00(97)	0.00(97)
MtArt+F	0.00(98)	0.00(98)	0.00(98)	0.00(98)	0.00(98)	0.00(98)	0.00(98)
MtREV+I	0.00(99)	0.00(99)	0.00(99)	0.00(99)	0.00(99)	0.00(100)	0.00(100)
MtREV	0.00(100)	0.00(100)	0.00(100)	0.00(100)	0.00(100)	0.00(99)	0.00(99)
HIVw+I+G	0.00(101)	0.00(101)	0.00(101)	0.00(101)	0.00(102)	0.00(102)	0.00(102)
HIVw+G	0.00(102)	0.00(102)	0.00(102)	0.00(102)	0.00(101)	0.00(101)	0.00(101)
HIVw+I	0.00(103)	0.00(103)	0.00(103)	0.00(103)	0.00(103)	0.00(103)	0.00(103)
MtMam+I+G	0.00(104)	0.00(104)	0.00(104)	0.00(104)	0.00(106)	0.00(106)	0.00(106)
MtMam+G	0.00(105)	0.00(105)	0.00(105)	0.00(105)	0.00(105)	0.00(105)	0.00(105)
HIVw	0.00(106)	0.00(106)	0.00(106)	0.00(106)	0.00(104)	0.00(104)	0.00(104)
MtArt+I+G	0.00(107)	0.00(107)	0.00(107)	0.00(107)	0.00(108)	0.00(108)	0.00(108)
MtArt+G	0.00(108)	0.00(108)	0.00(108)	0.00(108)	0.00(107)	0.00(107)	0.00(107)
MtMam+I	0.00(109)	0.00(109)	0.00(109)	0.00(109)	0.00(109)	0.00(109)	0.00(109)
MtArt+I	0.00(110)	0.00(111)	0.00(111)	0.00(110)	0.00(111)	0.00(111)	0.00(111)

```
MtMam           0.00(111)   0.00(110)   0.00(110)   0.00(111)   0.00(110)
0.00(110)   0.00(110)
MtArt           0.00(112)   0.00(112)   0.00(112)   0.00(112)   0.00(112)
0.00(112)   0.00(112)
---------------------------------------------------------------------------
---------------------------
Relative importance of
parameters      AIC         AICc-1      AICc-2      AICc-3      BIC-1
BIC-2       BIC-3
+G              0.12        0.16        0.14        0.12        0.44
0.51        0.75
+I              0.00        0.00        0.00        0.00        0.00
0.00        0.00
+I+G            0.88        0.84        0.86        0.88        0.56
0.49        0.25
+F              1.00        0.99        1.00        1.00        0.00
0.00        0.00
---------------------------------------------------------------------------
---------------------------
Model-averaged estimate of
parameters      AIC         AICc-1      AICc-2      AICc-3      BIC-1
BIC-2       BIC-3
alpha (+G)      4.49        4.49        4.49        4.49        4.64
4.64        4.64
p-inv (+I)      0.00        0.00        0.00        0.00        0.00
0.00        0.00
alpha (+I+G)    4.78        4.78        4.78        4.78        4.95
4.95        4.95
p-inv (+I+G)    0.00        0.00        0.00        0.00        0.00
0.00        0.00
---------------------------------------------------------------------------
---------------------------
AIC   : Akaike Information Criterion framework.
AICc-x: Second-Order Akaike framework.
BIC-x : Bayesian Information Criterion framework.
AICc/BIC-1: sample size as: number of sites in the alignment
(595.0)
AICc/BIC-2: sample size as: Sum of position's Shannon Entropy over the
whole alignment (1061.3)
AICc/BIC-3: sample size as: align. length x num sequences x averaged (0-
1)Sh. Entropy  (8839.9)
---------------------------------------------------------------------------
---------------------------


Save results to disk

Help about results in ProtTest's manual.
```

S2 Text. Protein sequences used for phylogenetic analysis

>OsDJ-1A
MAAQASPPTKKVLVPIVAGTEPVEAAVPIDVLRRAGADVTVASADDGELVVEVMYGVRIVADALVAGGDCAAAHFDLIVLPGGVPGAANLGGCA
ALEAMVRRHAATGGLYAAICAAPPLALASWGMLNGLKATAHPLFVDKFPPEVAAVDASVVVDASAVTSRGPATSTEFALALVEQLYSKNKAEQI
AKEMLVRYDAGYTIDEVNSVQWKCNGTPKVLVPVANGTEEMELITIIDVLRRADADVVVASAENAGVEIVARHGMRIVADTTLDEAAADDQTSS
FDLIILPGGTPGAKTMSSNEKLVTLLKKQAAASKPYGAIGAATAHVLEPHGLLEGKKAADQDGGDECESRVVVDGNVITSGGTGTAMEFAVAAV
EKLLGRDVAQRVAEGLLFA
>OsDJ-1B
MAMAAASASAMARRAASWPRLLLLSRAFAAAAAEPKRVLVPVADGTEPVEAAATADVLNRAGARVTVATADPAGDDRGLLVEAAFGVKLVADGR
VADLEGEAFDLIALPGGMPGSANLRDCKVLEKMVKKQAEQGGLYAAICATPAVTLAHWGLLKGLKATCYPSFMEKFTAEIIPVNSRVVVDRNAV
TSQGPATAIEYALALVEQLYGKEKSEEVAGPLYVRPQPGVDYVIDEFNSVEWKCSGTPQVLVPVANGSEEMEALNLIDILRRAGANVTVASVED
KLQVVTRRHKFNLIADIMVEEAAKREFDLIVMPGGLPGAQKLSSTKVLVDLLKKQAESNKPYGAICASPAYVLEPHGLLKGKKATSFPPMAHLL
TDQSACDSRVVVDGNLITSKAPGSATEFALAIVEKLFGREKAVSIAKELIFM
>OsDJ-1C
MAPKKVLLLCGDYMEDYEAMVPFQALQAYGVSVDAACPGKKAGDSCRTAVHQGIGHQTYAESRGHNFALNASFDEVNINEYDGLVIPGGRAPEY
LAMDEKVLDLVRKFSDAKKPIASVCHGQLILAAAGVVQNRKCTAYPAVKPVLVAAGAKWEEADTMDKCTVDGNLVTAVAYDAHPEFISLFVKAL
GGSVTGSNKRILFLCGDYMEDYEVMVPFQSLQALGCHVDAVCPDKGAGEKCPTAIHDFEGDQTYSEKPGHDFALTASFDNVDASSYDALVIPGG
RAPEYLALNDKVISLVKGFMDKAKPVASICHGQQILSAAGVLQGRKCTAYPAVKLNVVLGGATWLEPNPIDRCFTDGNLVTGAAWPGHPEFISQ
LMALLGIKVSF
>OsDJ-1D
MLPSSRYLLAPAPLPAMVVRPPPPHPPSRGTSPLARPPLCRAMARAAPSLSAAASTAASSSTTPAKKKVLLPIAMGTEEMEAVILAGVLRRAGA
DVTLASVEDGLEVEASRGSHIVADKRIAACADQVFDLVALPGGMPGSVRLRDSVILQRITVRQAEEKRLYGAICAAPAVVLMPWGLHKRKKITC
HPSFIEDLPTFRTVESNVQVSGELTTSRGPGTAFQFALSFVEQLFGPCKAEDMDNTLLTKVDDNLERSIEVNEIEWSSDHNPHVLIPIANGSEE
MEIIMLTDVLRRANVNVVLASVEKSTSIVGSQRMRIVADKCISDASALEYDLIILPGGPAGAERLHKSSVLKKLLKEQKQTGRMYGGICSSPVI
LQKQGLLQDKTVTAHPSIVNQLTCEVIDRSKVVIDGNLITGMGLGTVIDFSLAIIKKFFGHGRAKGVANGMVFEYPKS
>OsDJ-1E
MATRPLAASTLLPPLRFCSPLKTPPPSPPPPHLRRLQTLTRALASSSSAMASPPAKKVLVPIASGTEPMEAVITVDVLRRAGADVSVASVDPGS
AQVGGAWGVKLAADALLDDLADAEFDLISLPGGMPGSSNLRDCKLLENMVKKHAGKGKLYAAICAAPAVALGSWGLLNGLKATCYPSFMDKLPS
EVNAVESRVQIDGNCVTSRGPGTAMEYSVVLVEQLYGKEKADEVAGPMVMRPQHGVEFSLKELNSTSWNVGETPQILVPIANGTEEMEATMIID
ILRRAKANVVVASLEETLEIVASRKVKMVADVLLDDALKQQYDLILLPGGLGGAQAYAKSDKLIGLIKKQAEANKLYGAICASPAIALEPHGLL
KGKKATSFPGMWNKLSDQSECKNRVVVDGNLITSQGPGTSMEFSLAIVEKLFGRERAVELAKTMVFM
>OsDJ-1F
MAPCKKVLMLCGDYMEDYEAAVPFYALAAFGVAVDCVAPGKKPPGDACLTAVHEFLGHDLYTELPGHRFAVTADFAAAAAADASRYDALVVPGG
RFVERLSVDPLAVSLVAAFAGEGETATRRRPVVVTCHSQLLLAAAGAMRGVRCTAFFSMRRVVELAGGTWVEPDPLGLCVADGNVLSAIGWPAH
GEIIRELLRAMGARVAGGRGQAVLFLCADYVDDYEANVPFRALAGVGCRVEAACPTKRKGEACVTAIYDATPAAASDERRGHNFAVTADWGDVD
ADRYACVVVPGGRAPELLATRGEAVALVREFAGKGKVVASIDQGHLLLAAVGLLDGRSCASGVATRVVAGLAGAASVRHGGAVADGKLVTAASW
PDLAEFIAHIISLLGITVSF
>AtDJ-1B
MASSSLCHRYFNKITVTPFFNTKKLHHYSPRRISLRVNRRSFSISATMSSSTKKVLIPVAHGTEPFEAVVMIDVLRRGGADVTVASVENQVGVD
ACHGIKMVADTLLSDITDSVFDLIMLPGGLPGGETLKNCKPLEKMVKKQDTDGRLNAAICCAPALAFGTWGLLEGKKATCYPVFMEKLAACATA
VESRVEIDGKIVTSRGPGTTMEFSVTLVEQLLGKEKAVEVSGPLVMRPNPGDEYTITELNQVSWSFEGTPQILVPIADGSEEMEAVAIIDVLKR
AKANVVVAALGNSLEVVASRKVKLVADVLLDEAEKNSYDLIVLPGGLGGAEAFASSEKLVNMLKKQAESNKPYGAICASPALVFEPHGLLKGKK
ATAFPAMCSKLTDQSHIEHRVLVDGNLITSRGPGTSLEFALAIVEKFYGREKGLQLSKATLV
>AtDJ-1E
MASAVQKSALLLCGDYMEAYETIVPLYVLQSFGVSVHCVSPNRNAGDRCVMSAHDFLGLELTLNANFDDVTPENYDVIIIPGGRFTELLSADEK
CVDLVARFAESKKLIFTSCHSQVMLMAAGILAGGVKCTAFESIKPLIELSGGEWWQQPGIQSMFEITDCVKDGNFMSTVGWPTLGHGIKLLLES
LGGKVCSLEKKQASVLFLIGDYVEDYGINVPFRALQALGCKVDAVTPNKKKGEVCATAVYDLEDGRQIPAEKRGHNFFVTASWDDICVDDYDCV
VVPGGRSPELLVMNEKAVALVKSFAEKDKVFAAIGQGKLLLAATGVLKGKRCASGKGMKVMVKVAGGEAVMEKGCVTDGKVVTAASATDLPAFL
FDLSTALGLTVMF
>AtDJ-1D
MANSRTVLILCGDYMEDYEVMVPFQALQAFGITVHTVCPGKKAGDSCPTAVHDFCGHQTYFESRGHNFTLNATFDEVDLSKYDGLVIPGGRAPE
YLALTASVVELVKEFSRSGKPIASICHGQLILAAADTVNGRKCTAYATVGPSLVAAGAKWVEPITPDVCVVDGSLITAATYEGHPEFIQLFVKA
LGGKITGANKRILFLCGDYMEDYEVKVPFQSLQALGCQVDAVCPEKKAGDRCPTAIHDFEGDQTYSEKPGHTFALTTNFDDLVSSSYDALVIPG
GRAPEYLALNEHVLNIVKEFMNSEKPVASICHGQQILAAAGVLKGRKCTAYPAVKLNVVLGGGTWLEPDPIDRCFTDGNLVTGAAWPGHPEFVS
QLMALLGIQVSF
>AtDJ-1A
MASFTKTVLIPIAHGTEPLEAVAMITVLRRGGADVTVASVETQVGVDACHGIKMVADTLLSDITDSVFDLIVLPGGLPGGETLKNCKSLENMVK
KQDSDGRLNAAICCAPALALGTWGLLEGKKATGYPVFMEKLAATCATAVESRVQIDGRIVTSRGPGTTIEFSITLIEQLFGKEKADEVSSILLL
RPNPGEEFTFTELNQTNWSFEDTPQILVPIAEESEEIEAIALVDILRRAKANVVIAAVGNSLEVEGSRKAKLVAEVLLDEVAEKSFDLIVLPGG
LNGAQRFASCEKLVNMLRKQAEANKPYGGICASPAYVFEPNGLLKGKKATTHPVVSDKLSDKSHIEHRVVVDGNVITSRAPGTAMEFSLAIVEK
FYGREKALQLGKATLV
>AtDJ-1F
MGSMAQKSVLMLCGEFMEAYETIVPLYVLQAFGVSVHCVSPGRKTGDKCVMAAHDLLGLEIYTELVVDHLTLNANFDGVIPDQYDAIIIPGGRF
TELLSADEKCVSLVARFAELKKLIFTSCHSQLFLAAAGLLTGGMKCTAFESMKPFIELSGGAWWQQPGVQTLFEITDCVKDGSFMSTMGWPTLG
HSLKVLLESLGSKISSSKENHQTSLLFLIGDCVEDYSINVPFKAFQALGCKVDAVTPTKKRGEKCATIVHDLEDGRQLPTEKFGHNFYVTVAWD
DVSVDDYDCIVVPGGRSPELLVMNPKAVELVRKFVEKGKFVAAIGMGNWLLAATGALKKKRCASSYGTKVAVKVAGGEIVESERCVTDDKLVTA
ASTSDLPAFLYALSTALGLSVVF
>AtDJ-1C
MGSLGYSISMIASLSPTLMESRLISSMGCVSMTVAPSFSSVSVVSSSLGTTRRDRTLKLRSSMSPGMVTTLDSDVGVGSSATTKKVLVPIGYGT
EEIEAVVLVDVLRRAGADVTVASVEQKLEVEGSSGTRLLADVLISKCADQVYDLVALPGGMPGAVRLRDCEILEKIMKRQAEDKRLYGAISMAP
AITLLPWGLLTRKRTTGHPAFFGKLPTFWAVKTNIQISGELTTSRGPGTSFQFALSLAEQLFGETTAKSIEEFLLLRDGYQNPKNKEFNSIDWS
LDHTPRVLIPVANGSEAVELVSIADVLRRAKVDVTVSSVERSLRITAFQGTKIITDKLIGEAAESSYDLIILPGGHTGSERLQKSKILKKLLRE
QHESGRIYGATNSSSTVLHKHGLLKEKRTTVYPSESDEPMNQQMIEGAEVVIDGNVITSLGLATVTKFSLAIVSKLFGHARARSVSEGLVHEYP
RQ

>GmDJ-1C1.1
MHSINFKRQGGMPGSARLRDCDVLRKITCRQAEENSLYGAICAAPAVSLLPWGLLKKKKVSRGLTTSRGPGTSYQFALSLAEQLFGESVAKEVA
ELMLMRTDDDNAAKKEFNKVEWSVGHHTPSVLVPIVHGSEEIEVVTVVDILRRAKAKVIVASVEKSLEVLASQGTKIVADILIGDAQESPYDLI
ILPGGTAGAQRLSKSRILKKLLKEQNSAKRIYGAVYSSLAILQKQGLLKDKRTTAHPSVLVKLKDEEINGAKVDIDGKLITSEVLATVTDFALA
IVSKLFGNGRARSVAEGLVFEYPKECM
>GmDJ-1C2.1
MSLLLLPQPPTPLSTVTFSAAARAPFAAVTPPRPRTLTPKPALSLSAPITTTAPNNAIPPKKVLVPIGLGTEEMEAVIMIHVLRRAGADVTVAS
VEPQLQVEAAGGTKLVADTDISACSDQVFDLVALPWQGGMPGSARLRDCDVLRKITCRQAEENRLYGAICAAPAVTLLPWGLLKKKKITCHPAF
YDRLPRFWAVKSNLQVSRGLTTSRGPGTTYQFALSLAEQLFGDSVANEVAESMFMRTDDDHAAKEFNKVEWSVGHHTPSVLVPVAHGSEEIEVV
TVVDILRRAKAKVIVASVEKSLEVLASQGTKIVADILIGDAQESAHDLIILPGGTAGAQRLSKSRILKKLLKEQNSAERIYGAVCSSLAILQKQ
GLLKDKRATAHASTLDKLKDKEINGAKVVIDGKLITSEGLATVTDFALAIVSKLFGNGRARSVAEGLVFEYPKK
>GmDJ-1D1
MEDYEAMVPFQALQAFGLAIYPRKKSDDVCCTAIHVLADTQTYSETVGHNFALNATFDEVDASSYDGLWVPGGRAPEYLAHIPGVVELVTKFVS
LGKQIASICHGQLILAAAGVVEGRKCTLFLLLNQCWLLLAFGGKISGFDKKILFICGDYMEDYEVKDHFQSLQALGSHVDAVCPSKKAGDTCPT
AKPGHTFALTATFDDVDPSGYDALVIPGGQAPEYLALNESVIALILSAAGVLKGRKCSAYPAVKLNVVLSGAAWLEPESISRCFTDGNLVTGAA
WPGHPEFIAQLMALLGIQVSF
>GmDJ-1B
MALRHLRFFPHTLPLTLTPTPNPNNSNRFSFFTPSLSSTTLMATAHKVLVPIADGTEPMEAVITIDVLRRSGADVTVASASDNLAVQALHGVKI
IADAPVRDVAATSFDLVALPGGLQGVENLRDCKVLEGLVKKHVEDGRLYAAVCAAPAVVLGPWGLLNGKKATCYPALMEKLAAYAAATSESRVQ
VDGRVVTSRAPGTTMEFAITLIEQLIGKEKADEVAGPLVMHSNHDDEHTFKEFNPVQWTSDNPPKILVPIANGSEEMEAVIIIDILRRAKAKVV
VASVEDKLEIVASRKVKLEADMLLDEAAKLSYDLIVLPGGLGGAQTFANSETLVSLLKKQRESNIYYGAICASPALVLEPHGLLKGKKATAFPV
MCNKLSDQSEVENRVVVDGNLITSRGPGTSIEFALAIVEKLFGRKLALELAKAVVFARP
>GmDJ-1A
MALRHLRIFPHTLPLTLTPKPKLNNSNRFSFFTSSLSLSSTTLMATAHKVLVPIADGTEPMEAVIIIDVLRRSGADVTVASSSANLAVQALHGV
KIIADASVSDVAATAFDLVALPGGLQGDENLRDCKVLEGFVKKHVEDGRLYAAVCAAPAVVLGPWGLLNGKKATCYPALMEKLAAYVAATSESR
VQVDGTVVTSRAPGTTMEFAIALIEQLIGKEKAYEVAGPLVMRSNHDDEHTFKEFNSVQWTSDNPPKILVPIANGSEEMEAVIIIDILRRAKAK
VVVASVEDKLEIVASRKVKLEADMLLDEATKLSYDLIVLPGGLGGAQTFANSETLVSLLKKQRESNKYYGAICASPALVLEPHGLLKGKKATAF
PVMCDKLSDQSEVENRVVVDGNLITSRGPGTSIEFALAIVEKLFGRKLALELANAVVFARP
>GmDJ-1D2.1
MAPKKVLLLCGDFMEDYEAMVPFQALQAFGLAVDAVCPGKKSGDVCRTAVHVLAGAQTYSETVGHNFSLNATFDEVDAASYDGLWVPGGRAPEY
LAHVPGVVELVTKFVSLGKQIASICHGQLILAAAGVVKGRTCTAFPPVKPVLVAAGAHWVEPDTEAATVVDGDLITAATYEGHPELIRHFVKAL
GGKISGFDKKILFICGDYMEDYEVKVPFQSLQALGCHVDAVCPSKKAGDTCPTAVHDFEGDQTYSEKPGHTFALTATFDDVDPSGYDALVIPGG
RAPEYLALNESVIALVKYFFENKKPVASICHGQQILSAAGVLKGRKCTAYPAVKLNVVLSGATWLEPDPISRCFTDGNLVTGAAWPGHPEFIAQ
LIALLGIQVSF
>GmDJ-1D2.2
MAPKKVLLLCGDFMEDYEAMVPFQALQAFGLAVDAVCPGKKSGDVCRTAVHVLAGAQTYSETVGHNFSLNATFDEVDAASYDGLWVPGGRAPEY
LAHVPGVVELVTKFVSLGKQIASICHGQLILAAAGVVKGRTCTAFPPVKPVLVAAGAHWVEPDTEAATVVDGDLITAATYEGHPELIRHFVKAL
GGKISGFDKKILFICGDYMEDYEVKVPFQSLQALGCHVDAVCPSKKAGDTCPTAVHDFEGDQTYSEKPGHTFALTATFDDVDPSGYDALVIPGG
RAPEYLALNESVIALVKYFFENKKPVASICHGQQILSAAGVLKVLLL
>GmDJ-1D3
MASKRILLLCGDFTEDYEAMVPFQALQAFGLTVDTVCPGRKAGDVCRTAIHGIHGDQTYSEMIGHKFVLNATFDEVDASSYDVLWVPGGRSPEY
LSRVPGVLELVTKFVSLGKLIASTCHGPLILAASGVLKGRKCTGFPSLKPVLVDAGADWVDPDTMTTTVEDGGFITSTTYEGQPEIISLLVKAL
GGKISGTKKKILFICGDFVEDFQAKVPFQSLQSLGCHVDAICPSKFAGDFCPTAVHDFEGDQTYSEKHGHHFDLTVAFDDVDPSDYDALVIPGG
RSPEYLSLMDPILDLVRHFFLNNKPVGSIGHGQQILAAAGVLKGRKCTAYPDVKLHVVLSGATWLEPDPISRCFTDGNLVTGAAWQGLPEFIAQ
LMALLGIRVSF
>MtDJ-1A
MALSHIRFFPHTLPSTNFTPKLKLNHNRFFFSPSRSSSSSSSTITAMASNARKVLVPIADGTEPMEAVITIDVLRRSGADVTVASAANRLSVQA
LHGVKIIADASVSDVVNTAFDLVALPGGVPGVDNLRDSAVLEGLVKKHVEDGKLYAAVCAAPAVVLGPWGLLKGLKATGHPSFMEKLSSYTTSV
ESRVQLDGRVVTSRAPGTTMEFGVALVEQLLGKEKADEVAGPLVMRSNHADEYTFLELNSVQWTFDNPPKILVPIANGTEEMEAVIIVDILRRA
KANVVVASVEDKLEIEASRKVKLQADVLLDEAAKTSYDLIVLPGGIGGAQAFANSETLVNLLKKQRESNKYYGAICASPALALEPHGLLKGKKA
TGFPAMCSKLSDQSEVENRVVIDGNLITSRGPGTSIEFALVIVEKLFGRKLALEIANATVFASP
>MtDJ-1B
MKFCWFQTYIETVGHKFTLNRTFDEIDHTNLVDMLVNSGQEIIACICHGHMIQAAANLLEGPKCTAFPPLKLVLIAAGAFWFEHYVNNCSGW
>MtDJ-1C
MELEDMLVNSGQEIIACFCHGHLILAAANLLEGCKCTDFPPLKPVLIAAGAHWVEHLYLALFIALVKPFMENKKPVASICHSQHILAAAGVLKY
>MtDJ-1D
MAPKRVLLLCGDFMEDYEGMVPFQALQAFGVSVDAVCPGKKSGDVCRTAVHILSGGQTYTETVGHNFTLNATFDEVDHTSYDGLWLPGGRAPEY
LAHIPSVVELVTKFVKSGKEIACICHGHLILAAAGVVEGRKCTAFPPVKPVLVAAGAHWVEPDTMSTTVVDGNLITAPTYEGHPELLRHFLKAL
GGKISGSDKKILFICGDYMEDYEVKVPFQSLQALGCHVDAVCPSKKAGDTCPTAVHDFEGDQTYSEKPGHNFALTATFDDVDPSGYDALVIPGG
RSPEYLSLNEAVIALVKHFMENKKPVASICHGQQILAAAGVLKGRKCTAYPAVKLNVVLSGATWLEPDPISRCFTDGNLVTGAAWPGHPEFIAQ
LMALLGIQVSF
>MtDJ-1E
MSFLLLLLPQPSTATRLSPFTISTSTISLKPLSTLSPPRSIPNSTLSISTSPPPTPTNAPPPKKVLLPIGFGTEEMEAVILIHVLRRAGAHVTV
ASVEPQLQVEAASGTKLVADASISECSDQIFDLIALPGGMPGSARLRDCDALRIITCKQAEENRLFGAINAAPAVTLLPWGLLKRKKITCHPAF
FHKLPTFWAVKSNIQVSNGLTTSRGPGTAYMFALTLVEQLFGESIAREVAEFLLMRTDDDNVSKKEFNEIDWSVGHHPPSVLIPIAHGSEEIEV
VTLIDILRRAKANVVVASVEKTLGVMASQGTKIVADKLISDIQESAHDLIILPGGTAGAERLSKSRILKKLLKEQNSAGRIYGAVCSSPAILHK
QGLLKDKKATAHPSALNKLKDGAVNDAVVVIDGKVITSEGLATVTDFALAIVSKLFGNGRARSVAEGLVFEYPRK
>E.coli_Hsp31
MTVQTSKNPQVDIAEDNAFFPSEYSLSQYTSPVSDLDGVDYPKPYRGKHKILVIAADERYLPTDNGKLFSTGNHPIETLLPLYHLHAAGFEFEV
ATISGLMTKFEYWAMPHKDEKVMPFFEQHKSLFRNPKKLADVVASLNADSEYAAIFVPGGHGALIGLPESQDVAAALQWAIKNDRFVISLCHGP
AAFLALRHGDNPLNGYSICAFPDAADKQTPEIGYMPGHLTWYFGEELKKMGMNIINDDITGRVHKDRKLLTGDSPFAANALGKLAAQEMLAAYA
G
>Saccharomyces cerevisiae_Hsp31p
MAPKKVLLALTSYNDVFYSDGAKTGVFVVETLHPFNTFRKEGFEVDFVSETGKFGWDEHSLAKDFLNGQDETDFKNKDSDFNKTLAKIKTPKEV
NADDYQIFFASAGHGTLFDYPKAKDLQDIASEIYANGGVVAAVCHGPAIFDGLTDKKTGRPLIEGKSITGFTDVGETILGVDSILKAKNLATVE
DVAKKYGAKYLAPVGPWDDYSITDGRLVTGVNPASAHSTAVRSIDALKN
> Saccharomyces cerevisiae_Hsp32p

```
MTPKRALISLTSYHGPFYKDGAKTGVFVVEILRSFDTFEKHGFEVDFVSETGGFGWDEHYLPKSFIGGEDKMNFETKNSAFNKALARIKTANEV
NASDYKIFFASAGHGALFDYPKAKNLQDIASKIYANGGVIAAICHGPLLFDGLIDIKTTRPLIEGKAITGFPLEGEIALGVDDILRSRKLTTVE
RVANKNGAKYLAPIHPWDDYSITDGKLVTGVNANSSYSTTIRAINALYS
> Saccharomyces cerevisiae_Hsp33p
MTPKRALISLTSYHGPFYKDGAKTGVFVVEILRSFDTFEKHGFEVDFVSETGGFGWDEHYLPKSFIGGEDKMNFETKNSAFNKALARIKTANEV
NASDYKVFFASAGHGALFDYPKAKNLQDIASKIYANGGVIAAICHGPLLFDGLIDIKTTRPLIEGKAITGFPLEGEIALGVDDILRSRKLTTVE
RVANKNGAKYLAPIHPWDDYSITDGKLVTGVNANSSYSTTIRAINALYS
> Saccharomyces cerevisiae_Hsp34p
MTPKRALISLTSYHGPFYKDGAKTGVFVVEILRSFDTFEKHGFEVDFVSETGGFGWDEHYLPKSFIGGEDKMNFETKNSAFNKALARIKTANEV
NASDYKIFFASAGHGALFDYPKAKNLQDIASKIYANGGVIAAICHGPLLFDGLIDIKTTRPLIEGKAITGFPLEGEIALGVDDILRSRKLTTVE
RVANKNRAKYLAPIHPWDDYSITDGKLVTGVNANSSYSTTIRAINALYS
>Candida albicans_Hsp31
GSHMVKVLLALTSYNETFYSDGKKTGVFVVEALHPFEVFRKKGYEIQLASETGTFGWDDHSVVPDFLNGEDKEIFDNVNSEFNVALKNLKKASD
LDPNDYDIFFGSAGHGTLFDYPNAKDLQKIATTVYDKGGVVSAVCHGPAIFENLNDPKTGEPLIKGKKITGFTDIGEDILGVTDIMKKGNLLTI
KQVAEKEGATYIEPEGPWDNFTVTDGRIVTGVNPQSAVKTAEDVIAAFECN
>human_DJ-1
MASKRALVILAKGAEEMETVIPVDVMRRAGIKVTVAGLAGKDPVQCSRDVVICPDASLEDAKKEGPYDVVVLPGGNLGAQNLSESAAVKEILKE
QENRKGLIAAICAGPTALLAHEIGFGSKVTTHPLAKDKMMNGGHYTYSENRVEKDGLILTSRGPGTSFEFALAIVEALNGKEVAAQVKAPLVLK
DLE
>Drosophila melanogaster_DJ-1
GSHMSKSALVILAPGAEEMEFIIAADVLRRAGIKVTVAGLNGGEAVKCSRDVQILPDTSLAQVASDKFDVVVLPGGLGGSNAMGESSLVGDLLR
SQESGGGLIAAICAAPTVLAKHGVASGKSLTSYPSMKPQLVNNYSYVDDKTVVKDGNLITSRGPGTAYEFALKIAEELAGKEKVQEVAKGLLVA
YN
>Schizosaccharomyces pombe_DJ-1
MVKVCLFVADGTDEIEFSAPWGIFKRAEIPIDSVYVGENKDRLVKMSRDVEMYANRSYKEIPSADDFAKQYDIAIIPGGGLGAKTLSTTPFVQQ
VVKEFYKKPNKWIGMICAGTLTAKTSGLPNKQITGHPSVRGQLEEGGYKYLDQPVVLEENLITSQGPGTAMLFGLKLLEQVASKDKYNAVYKSL
SMP
>Schizosaccharomyces pombe_Hsp3101
MASEGKVLLVASSYYGPFYPDGMNTGVHFAELLIPYQVFREAGYEVQLTSETGKCKFDDHSIKKSALGEVERDAFDNKDNEFWYALKDIKPADK
INYKEFCIMFIAGGHAAMFDLPHATNLQTLAQQIYASNGVLAAVCHGPVMLPFVDDTKSPEGRSVVYGKKVTAFNSTGELVMGVSSALRERNMQ
DLNSLFREAGAEFVDPPTPMSDFTQVDGRIVTGVNPMSAKSTAEAAIKVSQSLRKT
>Schizosaccharomyces pombe_Hsp3102
MSIAKGKNALLVASSYYGPFYPDGKNTGVHFSELLIPYNVFKKAGFNVQFVSENGSYKFDDHSIEESKLGDFERKVFNDKNDDFWTNLNNMKKA
SDIVGKDYQLLFVAGGHAAMFDLPKATNLQAVAREVFTNGGVLSAVCHGPVLLANVKNPQSVEGKTVVYHKHVTAFNKAGEEKMGVMDELKKRG
MKSLNEIFAEAGATFIDPPNPNVNFTQIDGKIVTGVNPQSAKSTAEAAVSAL
>Caenorhabditis elegans_DJR-1.1
MAQKSALIIILAAEGAEEMEVIITGDVLARGEIRVVYAGLDGAEPVKCARGAHIVPDVKLEDVETEKFDIVILPGGQPGSNTLAESLLVRDVLKS
QVESGGLIGAICAAPIALLSHGVKAELVTSHPSVKEKLEKGGYKYSEDRVVVSGKIITSRGPGTAFEFALKIVELLEGKDKATSLIAPMLLKL
>Caenorhabditis elegans_DJR-1.2
MAAQKSALILLPPEDAEEIEVIVTGDVLVRGGLQVLYAGSSTEPVKCAKGARIVPDVALKDVKNKTFDIIIIPGGPGCSKLAECPVIGELLKTQ
VKSGGLIGAICAGPTVLLAHGIVAERVTCHYTVKDKMTEGGYKYLDDNVVISDRVITSKGPGTAFEFALKIVETLEGPEKTNSLLKPLCLAK
```
